# Supplementary material for: Targets, Mechanisms and Cytotoxicity of Half-Sandwich Ir(III) Complexes Are Modulated by Structural Modifications on the Benzazole Ancillary Ligand
Source: Cancers (Basel). 2022 Dec 24;15(1):107. doi: 10.3390/cancers15010107 (PMC9818021; doi:10.3390/cancers15010107)
Supplement: Supplementary file 1 [file cancers-15-00107-s001.zip › cancers-2057256-supplementary.pdf]

Supporting Information

Table of contents

1 - Characterization

Mass Spectra

1[C,NH-Cl]

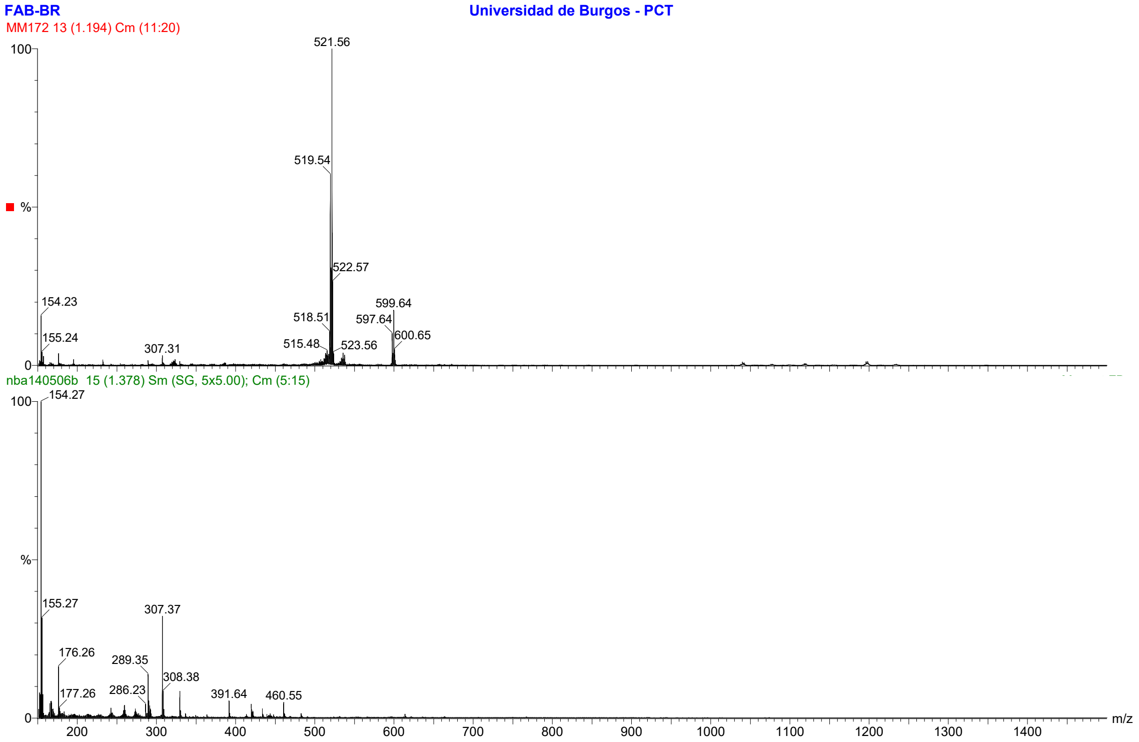

2[C,NMe-Cl]

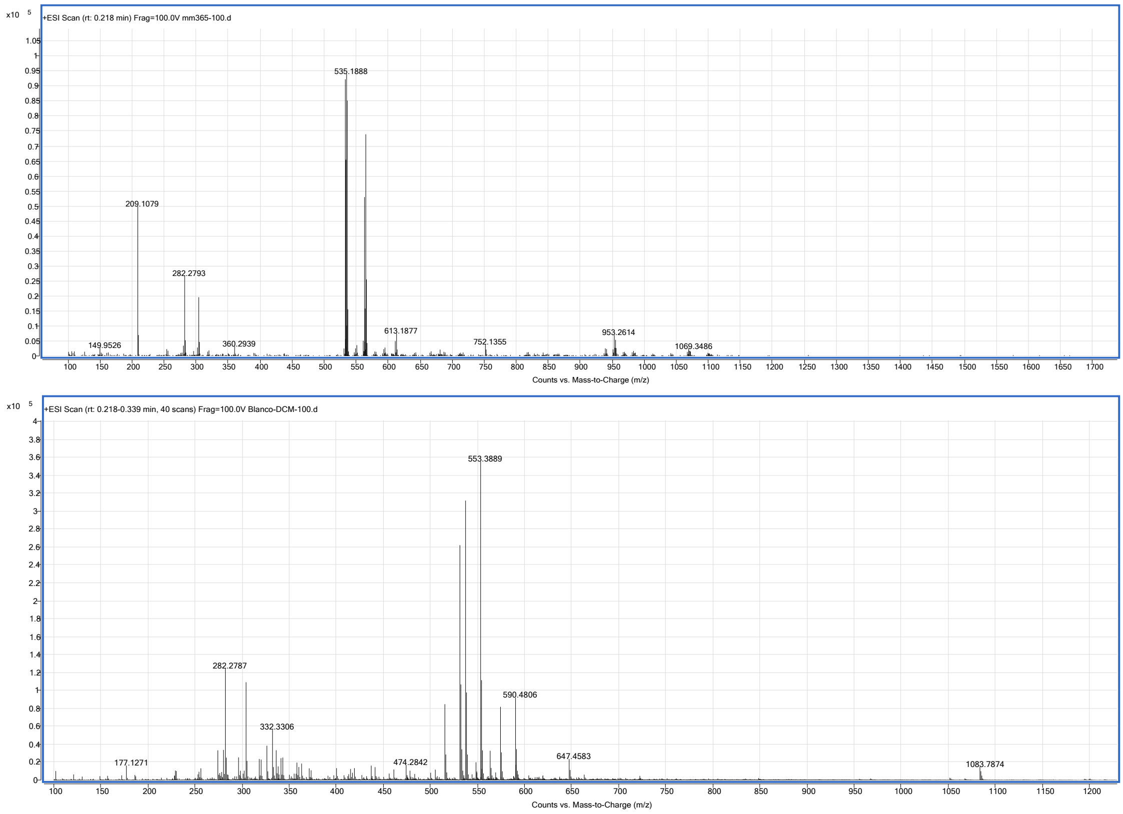

3[N,NH-Cl]Cl

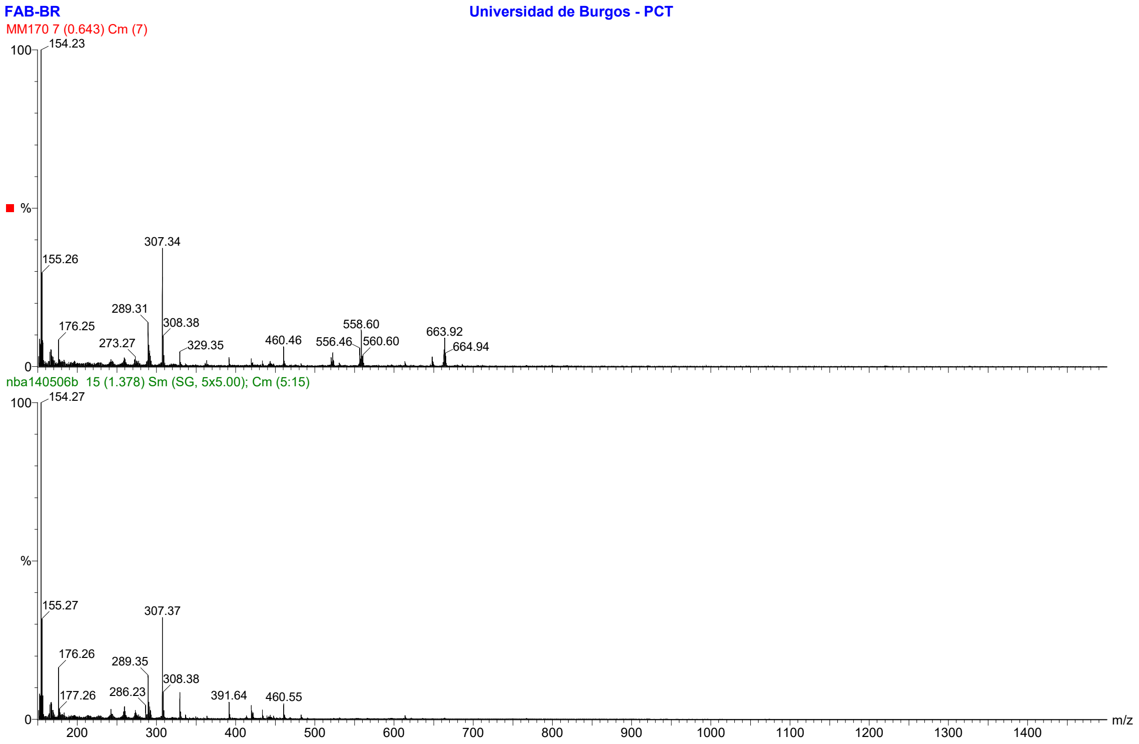

4[N,NMe-Cl]Cl

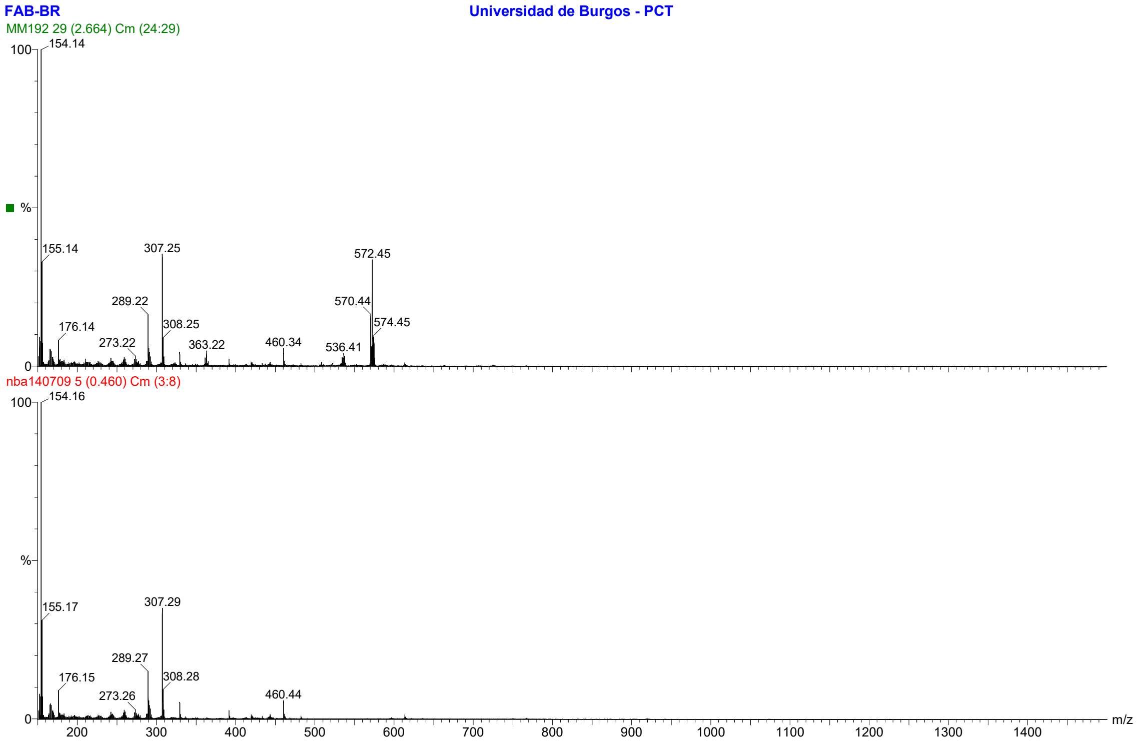

5[CNH_2,NH]Cl

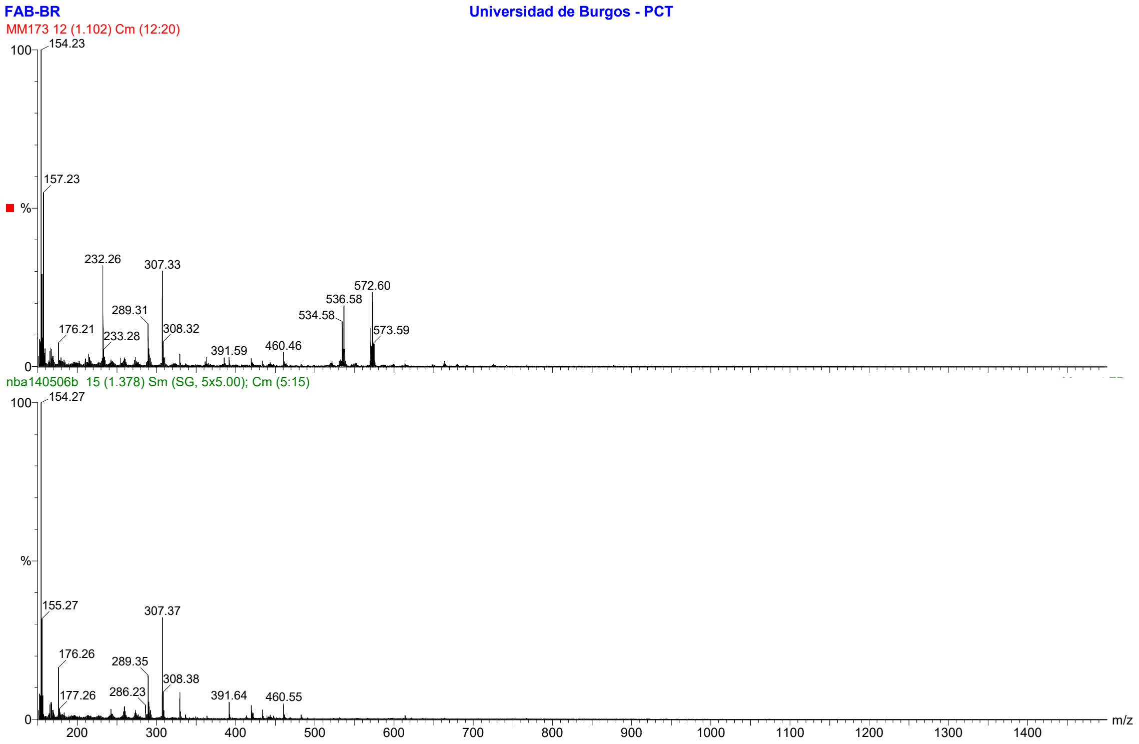

6[CNH_2,S-Cl]Cl

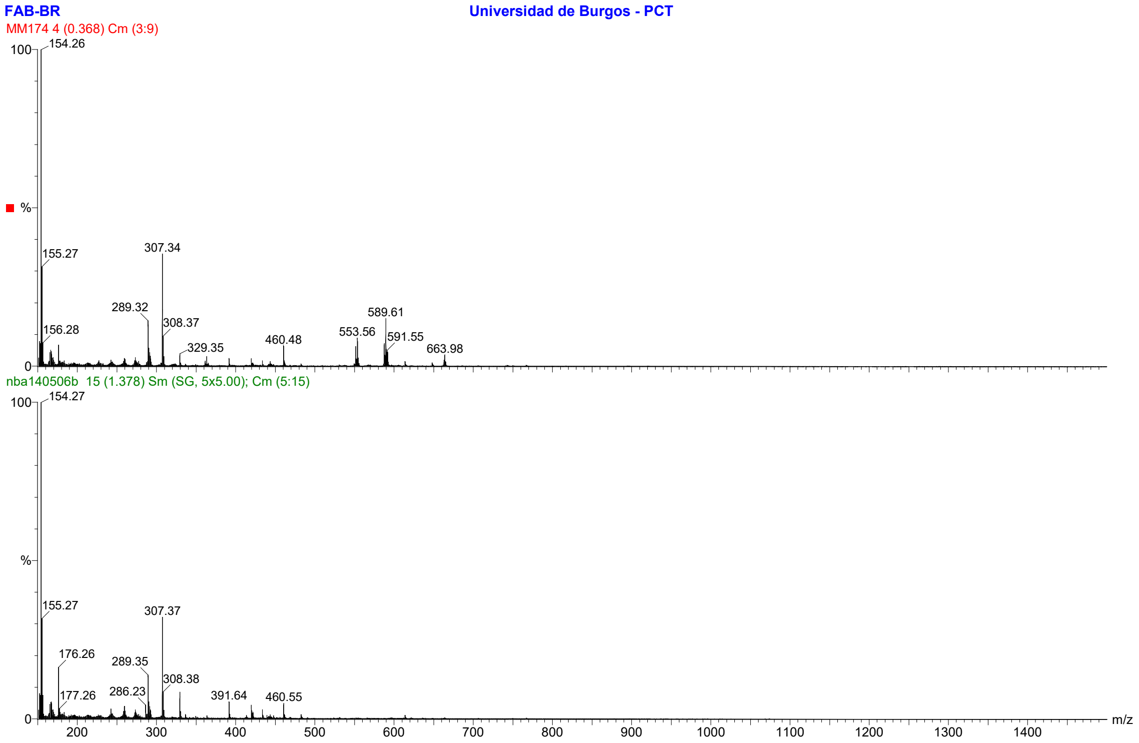

7[CO,NH-Cl]

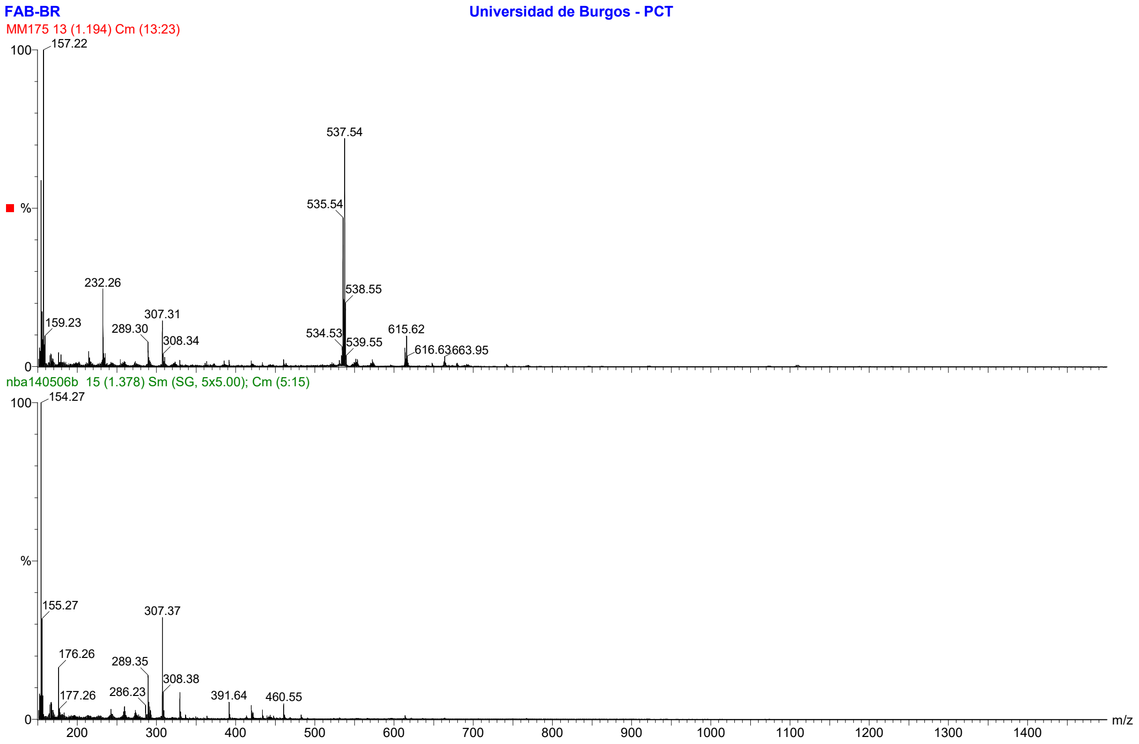

8[CO,S-Cl]

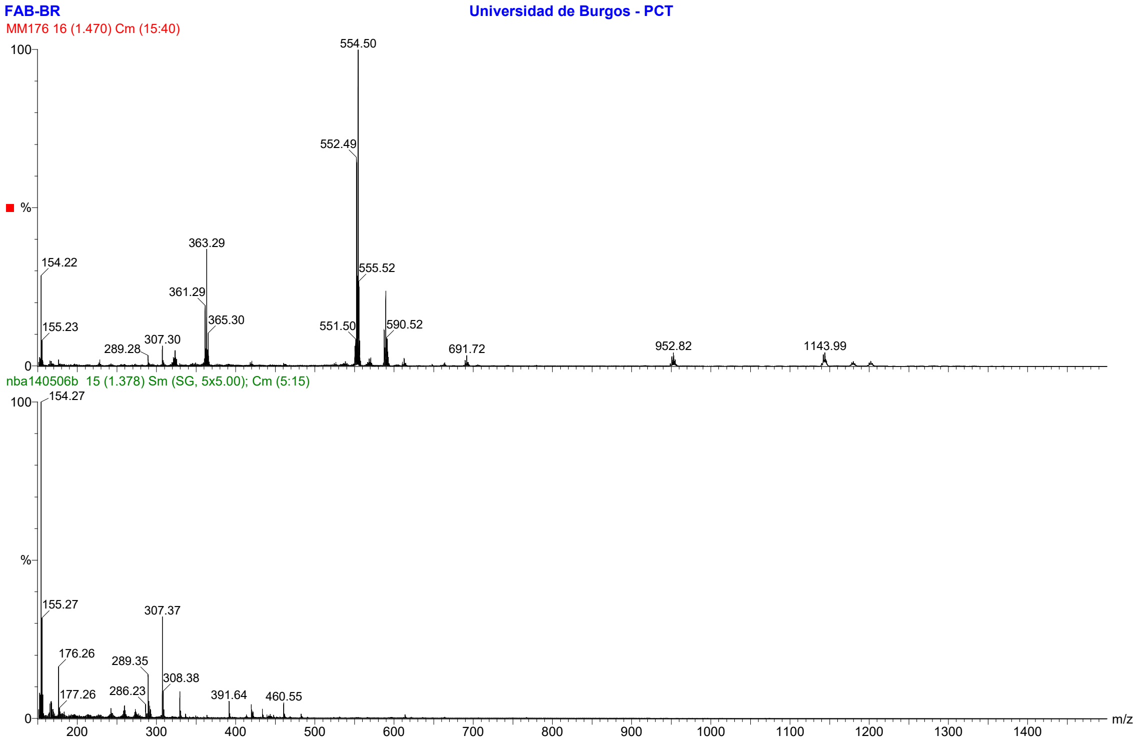

Figure S1. Mass spectra of the synthesized Ir(III) complexes.

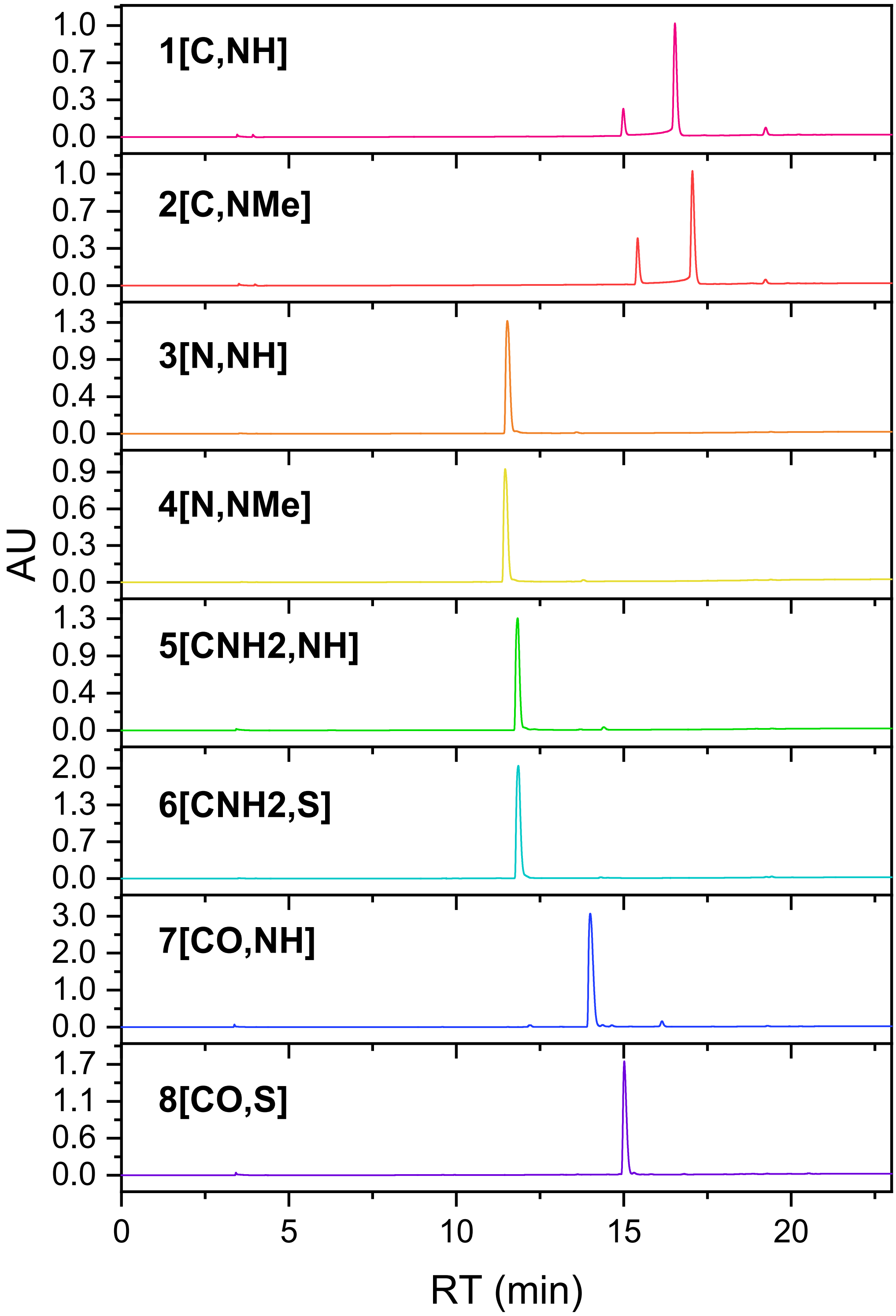

Figure S2. HPLC traces of lead compounds. As expected, the retention time (RT) of the neutral complexes is higher than those of the cationic ones. In addition, complexes **1[C,NH]** and **2[C,NMe]** display two peaks in the chromatogram, which are part of the equilibrium between the DMSO and acetonitrile species.

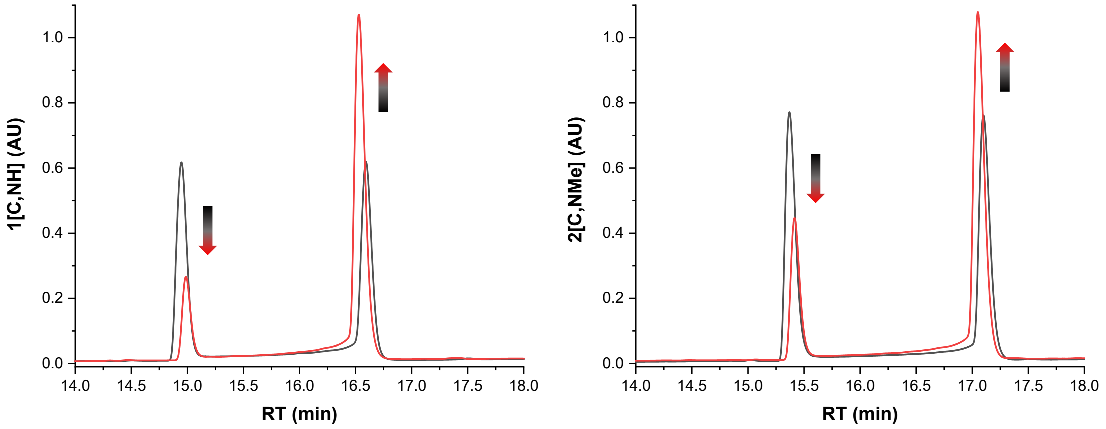

Figure S3. HPLC traces of complexes **1[C,NH]** and **2[C,NMe]** showing the equilibrium after overnight incubation at 50 °C. Recording time between first (black) and second (red) injections 3.5 h.

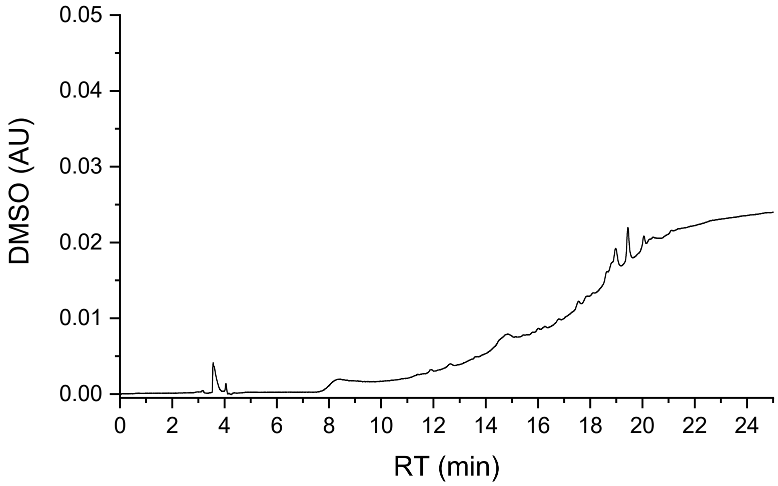

Figure S4. HPLC traces of dimethyl sulfoxide (DMSO).

Table S1. Selected bond lengths (Å) and angles (°) for complexes **1[C,NH-Cl]**, **3[N,NH-Cl]·2H$_2$O**, **4[N,NMe-Cl]·H$_2$O**, **5[CNH$_2$,NH-Cl]·H$_2$O** and **8[CO,S-Cl]**.

**Distance/ angle**	**1[C,NH-Cl]**	**Distance/ angle**	**3[N,NH-Cl] ·2H$_2$O**	**4[N,NMe-Cl] ·H$_2$O**
**Ir1-Cl1/ Ir2-Cl2**	2.422(4)/2.415(4)	Ir1-Cl1	2.3914(12)	2.4048(11)
**Ir1-C1/Ir2-C31**	2.13(2)/2.202(11)	Ir1-N2	2.088(3)	2.072(3)
**Ir1-N1/Ir2-N3**	2.072(17)/2.037(16)	Ir1-N1	2.136(4)	2.097(4)
**N1-C7/N3-C37**	1.34(2)/1.33(3)	N2-C6	1.345(5)	1.329(5)
**N2-C7/N4-C37**	1.34(2)/1.33(3)	N3-C6	1.336(6)	1.364(5)
**C1-Ir1-N1/ C31-Ir2-N3**	76.6(8)/75.9(7)	N1-Ir1-N2	78.28(14)	75.69(14)
**N1-Ir1-Cl1/ N3-Ir2-Cl2**	85.2(5)/85.9(5)	N1-Ir1-Cl1	87.41(10)	87.48(10)
**C1-Ir1-Cl1/**	92.1(5)/89.5(4)	N2-Ir1-Cl1	84.29(10)	85.50(10)

C31-Ir2-Cl2			

Distance/ angle	5[CNH_2,NH-Cl]·H_2O	Distance/ angle	8[CO,S-Cl]
**Ir1-Cl1**	2.422(12)	**Ir1-Cl1**	2.3847(11)
**Ir1-N2**	2.090(4)	**Ir1-N1**	2.098(4)
**Ir1-N1**	2.140(4)	**Ir1-O1**	2.110(3)
**N1-C1**	1.456(6)	**O1-C1**	1.312(6)
**N2-C7**	1.331(6)	**N1-C7**	1.311(6)
**N3-C7**	1.348(6)	**S1-C7**	1.741(5)
**N1-Ir1-N2**	80.34(15)	**O1-Ir1-N1**	81.77(14)
**N1-Ir1-Cl1**	83.14(12)	**O1-Ir1-Cl1**	85.20(10)
**N2-Ir1-Cl1**	84.35(11)	**N1-Ir1-Cl1**	86.62(11)

Table S2. Selected geometric parameters[a] for the metal complexes of **1[C,NH-Cl]**, **3[N,NH-Cl]·2H_2O**, **4[N,NMe-Cl]·H_2O**, **5[CNH_2,NH-Cl]·H_2O** and **8[CO,S-Cl]**.

Distance/angle	1[C,NH-Cl]	3[N,NH-Cl] ·2H_2O	4[N,NMe-Cl] ·H_2O	5[CNH_2,NH-Cl] ·H_2O	8[CO,S-Cl]
**Range Ir–C distances**	2.157(18)-2.23(2)/ 2.149(17)-2.27(2)	2.153(4)-2.179(4)	2.142(4)-2.183(4)	2.132(4)-2.167(5)	2.137(4)-2.154(4)
**Ir–centroid**	1.825/1.818	1.790	1.782	1.774	1.766
**α^b**	6.19/4.89	11.75	22.55	30.95	27.27
**θ (N-C-C-X)^c (X=N,C)**	4.93/2.86	-2.33	-2.09	30.47	25.08
**β (chelate-Cp*)^d**	56.63/55.13	49.39	46.66	14.73	15.81
**λ^e**	0.67/1.82	6.74	11.14	44.79	42.87

[a]Calculated with Mercury, version 4.3. [b]α = Angle between the mean planes of the two rings in the bidentate ligand, pyridyl or and diazole. [c]Θ = Torsion angle formed by the atoms of the chelate ring (N-C-C-X). [d]β = Angle between the mean planes of the chelate ring (defined by the four atoms of the ligand) and the arene. [e]λ = Angle between the mean planes formed by N-Ru-X and N-C-C-X (X = N or C), as a measure of the planarity of the chelate ring.

Table S3. Crystal data and structure refinement for **1[C,NH-Cl]**, **3[N,NH-Cl]·2H_2O** and **4[N,NMe-Cl]·H_2O**.

	1[C,NH]	3[N,NH-Cl]·2H_2O	4[N,NMe-Cl]·H_2O
**Empirical formula**	$C_{23}H_{24}ClIrN_2$	$C_{22}H_{28}Cl_2IrN_3O_2$	$C_{23}H_{28}Cl_2IrN_3O$
**Formula weight**	556.09	629.57	625.58
**Temperature (K)**	290(2)	290(2)	173(2)
**Wavelength (Å)**	0.71073	0.71073	0.71073
**Crystal system**	Monoclinic	Monoclinic	Monoclinic
**Space group**	P 2_1/c	P 2_1/c	P 2_1/c
**a (Å)**	7.5060(16)	17.356(4)	18.514(3)
**b (Å)**	18.879(4)	7.4700(17)	7.4111(13)
**c (Å)**	27.757(6)	18.290(5)	17.866(3)
**α (°)**	90	90	90
**β (°)**	90.309(4)	93.749(4)	112.039(3)
**γ (°)**	90	90	90
**Volume ($Å^3$)**	3933.2(15)	2366.2(10)	2272.3(7)
**Z**	8	4	4

**Density (calculated) (g/cm³)**	1.878	1.767	1.829
**Absorption coefficient (mm⁻¹)**	6.935	5.891	1.829
**F(000)**	2160	1232	1224
**Crystal size (mm³)**	0.21x0.11x0.10	0.29 x 0.11 x 0.10	0.31 x 0.22 x 0.11
**Theta range for data collection (°)**	1.304 to 24.997	2.232 to 24.999	1.187 to 28.228
**Index ranges**	-8≤h≤8, -22≤k≤22, -33≤l≤33	-20≤h≤20, -8≤k≤8, -21≤l≤21	-24≤h≤24, -9≤k≤9, -23≤l≤22
**Reflections collected**	37911	21956	25433
**Independent reflections**	6906 [R(int) = 0.0924]	4073 [R(int) = 0.0326]	5286 [R(int) = 0.1235]
**Completeness to theta = 25.00°**	100.0 %	95.3 %	100.0 %
**Absorption correction**	Semi-empirical from equivalents	Semi-empirical from equivalents	Semi-empirical from equivalents
**Max. and min. transmission**	0.746 and 0.543	1.000 and 0.346	0.746 and 0.406
**Refinement method**	Full-matrix least-squares on F2	Full-matrix least-squares on F2	Full-matrix least-squares on F2
**Data / restraints / parameters**	6906 / 600 / 461	4073 / 0 / 280	5286 / 0 / 277
**Goodness-of-fit on F2**	1.129	1.051	1.023
**Final R indices [I>2sigma(I)]**	R1 = 0.0932, wR2 = 0.1931	R1 = 0.0258, wR2 = 0.0593	R1 = 0.0387, wR2 = 0.0832
**R indices (all data)**	R1 = 0.1176, wR2 = 0.2040	R1 = 0.0285, wR2 = 0.0604	R1 = 0.0423, wR2 = 0.0933
**Largest diff. peak and hole, e·Å⁻³**	7.699 and -4.207	2.385 and -0.610	4.127 and -2.482

Table S4. Crystal data and structure refinement for **5[CNH$_2$,NH-Cl]·H$_2$O** and **8[CO,S-Cl].**

	**5[CNH$_2$,NH-Cl]·H$_2$O**	**8[CO,S-Cl]**
**Empirical formula**	$C_{23}H_{27}Cl_2IrN_3O_{0.5}$	$C_{23}H_{23}ClIrNOS$
**Formula weight**	616.57	589.13
**Temperature (K)**	290(2)	293(2)
**Wavelength (Å)**	0.71073	0.71073
**Crystal system**	Monoclinic	Monoclinic
**Space group**	C 2/c	P 2_1/c
**a (Å)**	39.176(5)	17.492(2)
**b (Å)**	7.2131(8)	7.1040(10)
**c (Å)**	17.803(2)	17.238(2)
**α (°)**	90	90
**β (°)**	115.546(2)	103.360(2)
**γ (°)**	90	90
**Volume (Å³)**	4539.0(9)	2084.1(5)
**Z**	8	4

**Density (calculated) (g/cm^3)**	1.805	1.878
**Absorption coefficient (mm^{-1})**	6.136	6.649
**F(000)**	2408	1144
**Crystal size (mm^3)**	0.390 x 0.210 x 0.180	0.300 x 0.110 x 0.100
**Theta range for data collection (°)**	2.289 to 24.999	2.393 to 24.999
**Index ranges**	-46≤h≤46, -8≤k≤8, -21≤l≤21	-20≤h≤20, -8≤k≤8, -20≤l≤20
**Reflections collected**	21497	19814
**Independent reflections**	4009 [R(int) = 0.0409]	3684 [R(int) = 0.0302]
**Completeness to theta = 25.00°**	97.6 %	97.5 %
**Absorption correction**	Semi-empirical from equivalents	-
**Max. and min. transmission**	1.000 and 0.512	-
**Refinement method**	Full-matrix least-squares on F2	Full-matrix least-squares on F2
**Data / restraints / parameters**	4009 / 2 / 288	3684 / 0 / 258
**Goodness-of-fit on F2**	0.993	1.023
**Final R indices [I>2sigma(I)]**	R1 = 0.0253, wR2 = 0.0632	R1 = 0.0257, wR2 = 0.0608
**R indices (all data)**	R1 = 0.0296, wR2 = 0.0652	R1 = 0.0267, wR2 = 0.0614
**Largest diff. peak and hole, e·Å^{-3}**	1.450 and -0.873	3.283 and -0.846

Molar conductivity experiments in acetonitrile also confirmed the nature and charge of the complexes (see table S5) and were performed in acetonitrile (10^{-3} M) for all the complexes, except for **1[C,NH-Cl]** and **2[C,NMe-Cl]**, due to their low solubility. The results allowed us to extract some conclusions. Among the cationic complexes, all those with the benzimidazole moiety present extremely lower values (27.1 – 50.6 S·cm^2·mol^{-1}) than those with benzothiazole. The NH group is prone to participating in hydrogen bonding interactions, promoting the formation of ion-pairing with chloride, which is favoured in non-polar solvents of low dielectric constant or in a protic solvent. Dielectric constants are key parameters to determine ion-pairing. The complexes with benzothiazole moieties (79.6 – 89.4S·cm^2·mol^{-1}) have intermediate values, as they are also able to form ion pairing; **6[CNH$_2$,S-Cl]** through the NH_2 group. Nonetheless, in complex **4[N,NMe-Cl]** (140.9 S·cm^2·mol^{-1}) the N-Me unit prevents the N atom from taking part in hydrogen bonding interactions. Therefore, its molar conductivity value is in the normal range. Regarding the neutral complexes and just as expected, they have the lowest conductivities (10.5 – 19.0 S·cm^2·mol^{-1}).

Table S5. Molar conductivity values for complexes measured in acetonitrile (10^{-3} M).

Complex	Overall charge	Λ_m (S·cm^2·mol^{-1})
**1[C,NH-Cl]**	0 / neutral	insoluble
**2[C,NMe-Cl]**	0 / neutral	insoluble
**3[N,NH-Cl]**	1 / monocationic	27.1
**4[N,NMe-Cl]**	1 / monocationic	140.9

**5[CNH$_2$,NH-Cl]**	1 / monocationic	50.6
**6[CNH$_2$,S-Cl]**	1 / monocationic	79.6
**7[CO,NH-Cl]**	0 / neutral	Insoluble
**8[CO,S-Cl]**	0 / neutral	10.5

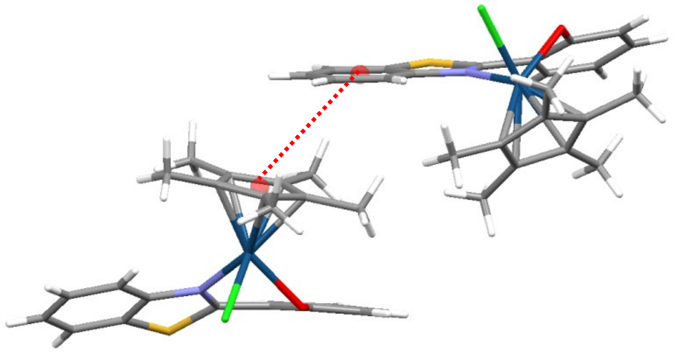

Figure S5. π-π stacking between Cp* and the benzothiazole moiety in **8[CO,S]**.

Table S6. π-π offset staking parameters for complex **8[CO,S]**.

Compound	$d_{cent\text{-}cent}$ (Å)	α (°)	$d_{cent\text{-}pl}$ (Å)	β (°)	d_{offset} (Å)
**8[CO,S-Cl]**	4.428	12.11	3.063	46.23	3.198
			3.661	34.23	2.491

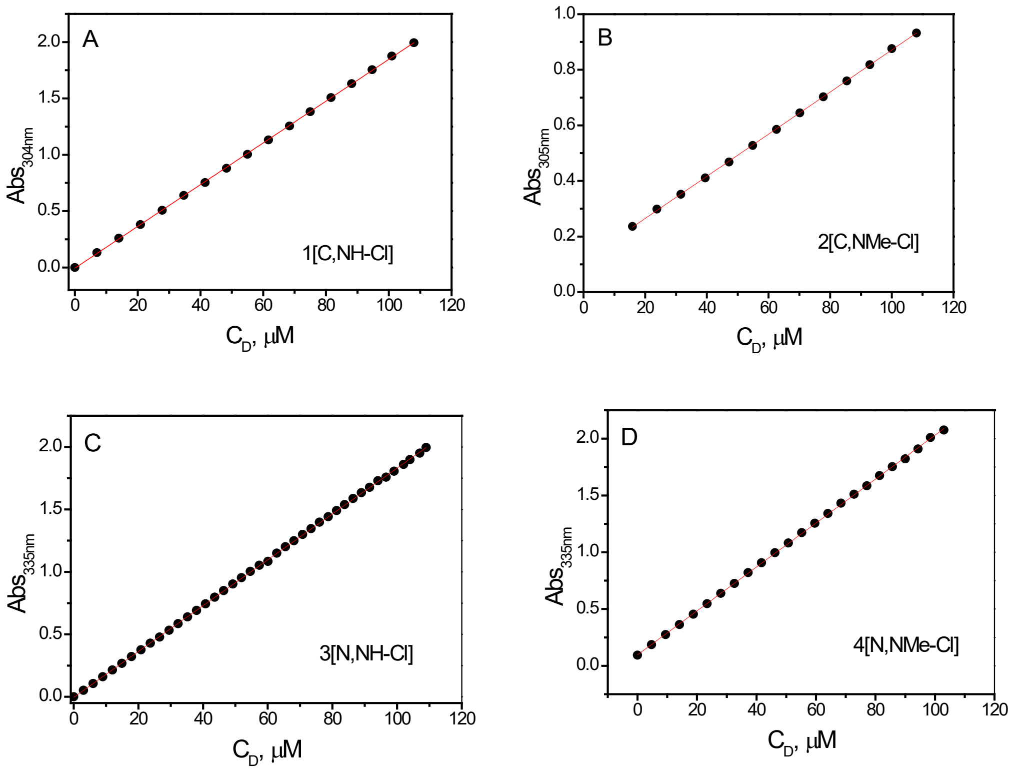

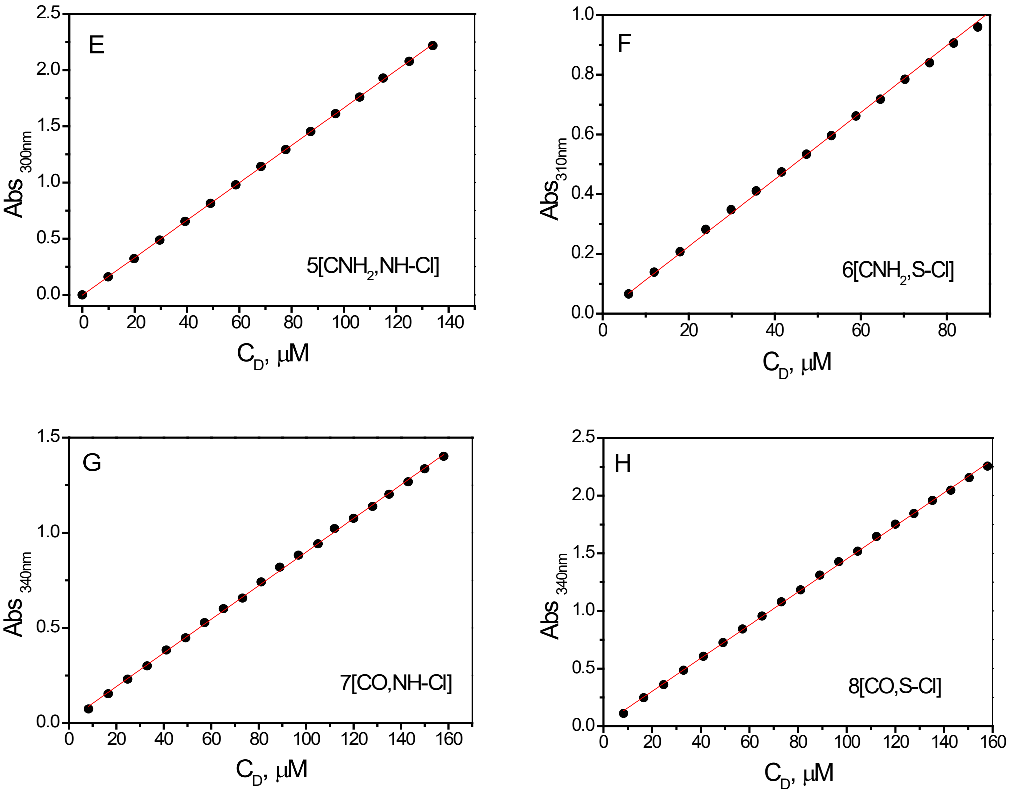

Figure S6. Fulfilment of Lambert-Beer law in 2.5 mM NaCaC buffer, pH = 7 and T = 25 °C. A) **1[C,NH-Cl]**, B) **2[C,NMe-Cl]**, C) **3[N,NH-Cl]**, D) **4[N,NMe-Cl]**, E) **5[CNH$_2$,NH-Cl]**, F) **6[CNH$_2$,S-Cl]**, G) **7[CO,NH-Cl]** and H) **8[CO,S-Cl]**

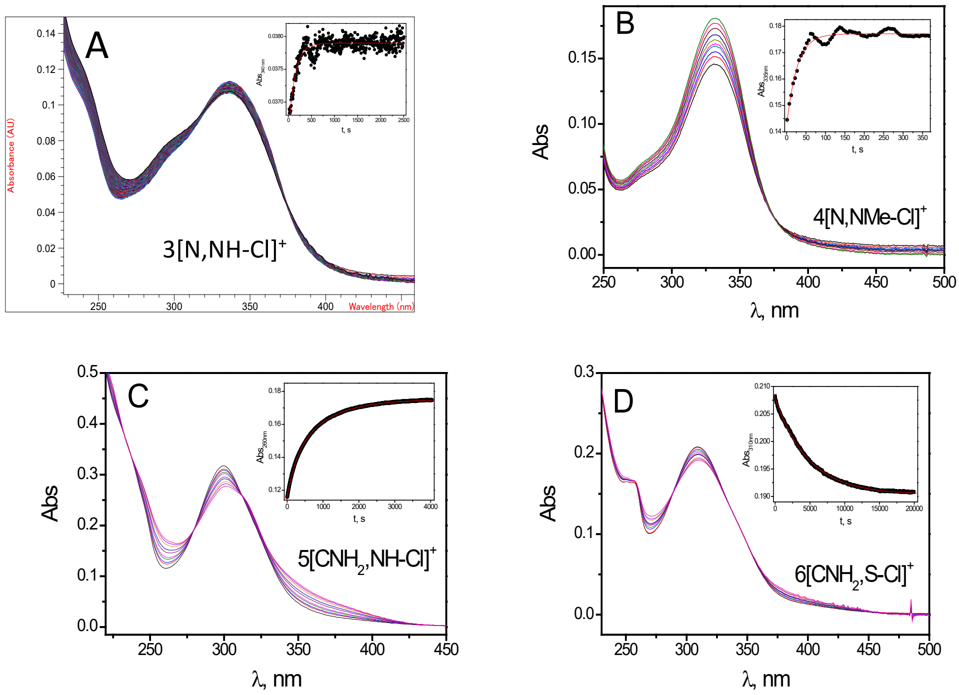

Figure S7. Recorded spectra of aquation processes and the corresponding absorbance *versus* time plot at a fixed λ; monoexponential fitting curves (red lines): A) **3[N,NH-Cl]$^+$**, B) **4[N,NMe-Cl]$^+$**, C) **5[CNH$_2$,NH-Cl]$^+$**, D) **6[CNH$_2$,S-Cl]$^+$**. C_D = 1.5 x 10^{-5} M.

Table S7. Rate constants for the Cl-complexes reaction to form H_2O-complexes (k_{aq}) or DMSO-complexes (k_{DMSO}).

**Complex**	**k_{aq}, s^{-1}**	**K_{DMSO}, s^{-1}**
**1[C,NH-Cl]**	-	$(1.79 \pm 0.04) \times 10^{-3}$
**2[C,NMe-Cl]**	-	$(6.63 \pm 0.01) \times 10^{-3}$
**3[N,NH-Cl]$^+$**	$(7.11 \pm 0.32)\times 10^{-3}$	-
**4[N,NMe-Cl]$^+$**	$(3.81 \pm 0.19)\times 10^{-2}$	-
**5[CNH$_2$,NH-Cl]$^+$**	$(1.47 \pm 0.01) \times 10^{-3}$	-
**6[CNH$_2$,S-Cl]$^+$**	$(2.12 \pm 0.01) \times 10^{-4}$	-
**7[CO,NH-Cl]**	-	$(7.67 \pm 0.51) \times 10^{-3}$
**8[CO,S-Cl]**	-	$(3.11 \pm 0.25) \times 10^{-4}$

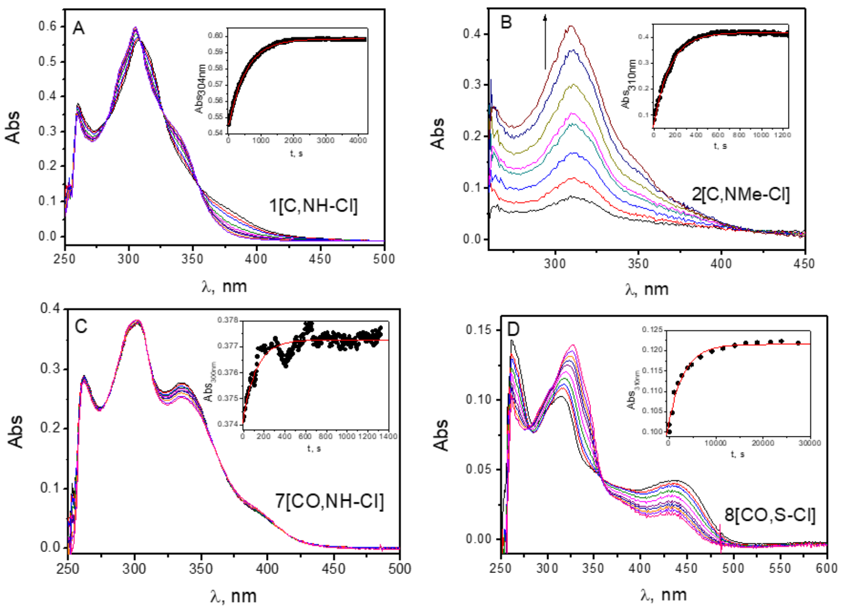

Figure S8. Spectral profiles over time of the [complex-DMSO]$^+$ formation for A) **1[C,NH-Cl]** B) **2[C,NMe-Cl]** C) **7[CO,NH-Cl]** and D) **8[CO,S-Cl]**. Inserts: Monoexponential fitting curves corresponding to absorbance *versus* time at a fixed λ (red lines).

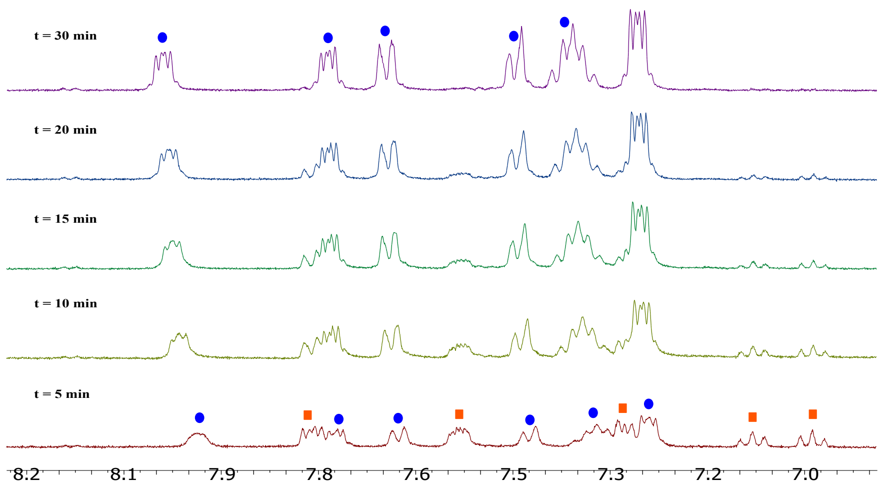

Figure S9. ^{1}H NMR spectra for the substitution reaction of chloride by DMSO-d_6 in complex **1[C,NH-Cl].** The orange squares depict the original complex **1[C,NH-Cl],** whereas the blue spots correspond to the DMSO derivative **1[C,NH-DMSO].**

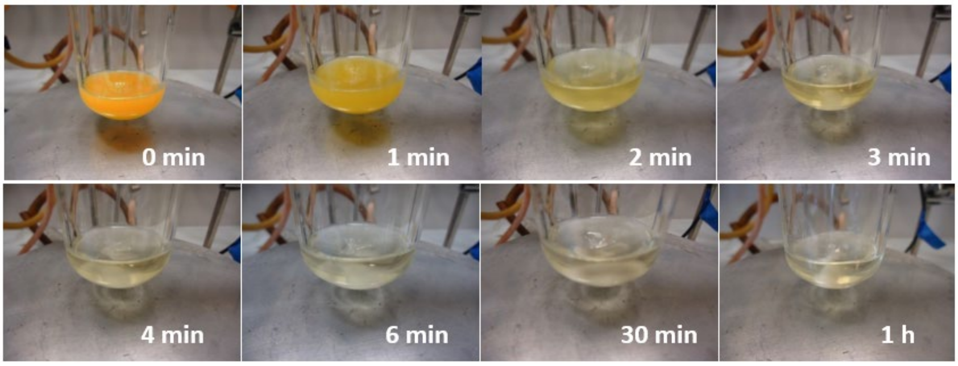

Figure S10. Evolution of a solution of **1[C,NH-Cl]** in DMSO with time. The suspension gets colourless as the substitution evolves.

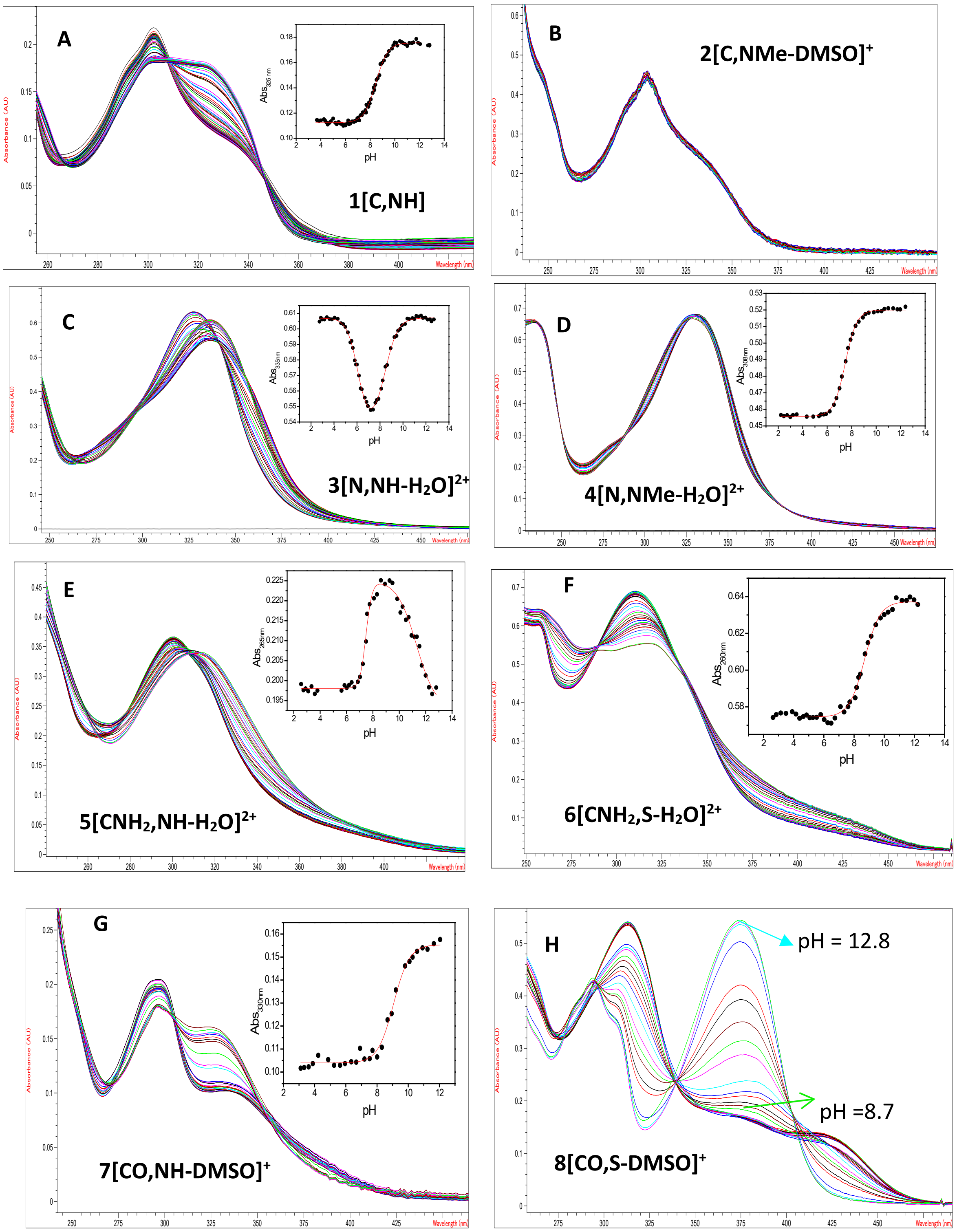

Figure S11. UV vis spectrograms and absorbance at different λ as a function of pH (insets). λ = 235 (A), 335 (B), 308 (C), 265 (D), 260 (E), 330 (F) and 375 nm (G).

Table S8. Dissociation acidity constants of the studied iridium complexes. pK_{a1} corresponds to water dissociation to give the corresponding hydroxo complex and pK_{a2} correspond to NH dissociation to give the N anion.

Complex	pK_{a1}	pK_{a2}
1[C,NH-DMSO]$^+$	-------	8.44 ± 0.03
2[C,NMe-DMSO]$^+$	-------	------
3[N,NH-H_2O]$^{2+}$	6.08 ± 0.04	8.44 ± 0.06
4[N,NMe-H_2O]$^{2+}$	7.48 ± 0.01	-------
5[CNH_2,NH-H_2O]$^{2+}$	7.48 ± 0.04	11.30 ± 0.17
6[CNH_2,S-H_2O]$^{2+}$	8.70 ± 0.04	-------
7[CO,NH-DMSO]$^+$	-------	9.06 ± 0.06
8[CO,S-DMSO]$^+$	-------	-------

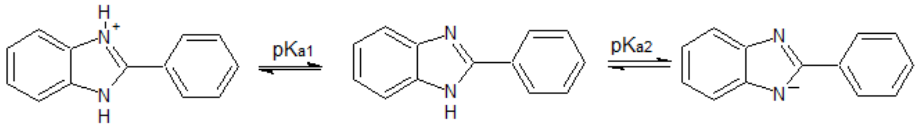

Scheme S1: Ionization equilibria of 2-phenylbenzimidazole ligand. $pKa_1 = 5.2 \pm 0.1$ y $pKa_2 = 11.8 \pm 0.1$

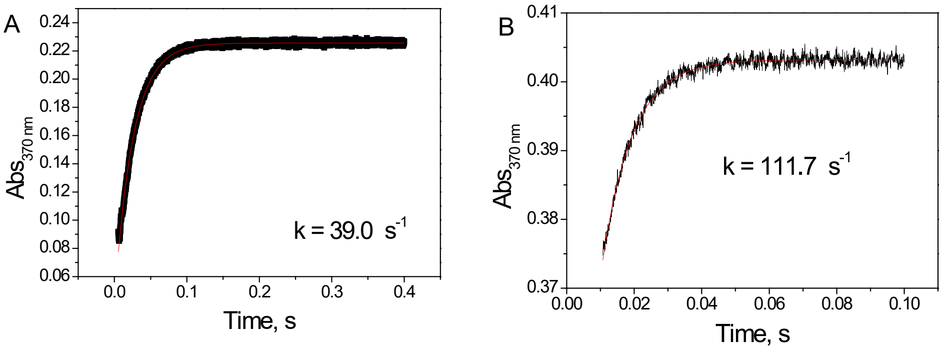

Figure S12. Stopped flow kinetic curves for **8[CO,S]** recorded at pH = 10.2 (A) and at pH =11.5 (B). The red line shows the fitting of a single exponential function to the Abs-Time data pairs.

Table S9. Cytotoxic activity in ovarian A2780, A2780cis (ovarian acquired resistance to cisplatin), colon SW480 and lung A549 cancer cells of complexes **1** - **8** at 48 h expressed as IC_{50} values in µM.

	**A2780**	**A2780CIS**	**SW480**	**A549**
**1[C,NH]**	5 ± 2	5 ± 1	5 ± 2	10 ± 1
**2[C,NMe]**	9 ± 1	13 ± 1	61 ± 1	10 ± 1
**3[N,NH]**	84 ± 1	48 ± 1	90 ± 1	> 100
**4[N,NMe]**	>100	>100	>100	> 100
**5[CNH_2,NH]**	17 ± 1	30 ± 1	34 ± 1	>100
**6[CNH_2,S]**	>100	48 ± 2	40 ± 1	>100
**7[CO,NH]**	14 ± 1	42 ± 1	>100	43 ± 3
**8[CO,S]**	2 ± 1	6 ± 2	34 ± 1	41 ± 5
**cisPt**	7 ± 1	60 ± 1	34 ± 1	33 ± 6

2 - Interaction with DNA

Kinetic study

Aquo-complexes bind DNA(N7) more easily than their chlorido analogous[1],[2]. Hence, all complexes were incubated overnight until the aquation process is completed. Water-insoluble compounds were firstly dissolved in DMSO and then diluted in water.

From the fitting of absorbance-time graphs (Figure S8) to a biexponential analysis, two rate constants were obtained. The fast reaction could be related to covalent binding, being k_c the rate constants (Table S9), while the slow reaction does not follow any pattern and it is associated to DNA/Ir-complex rearrangements, so it was disregarded.

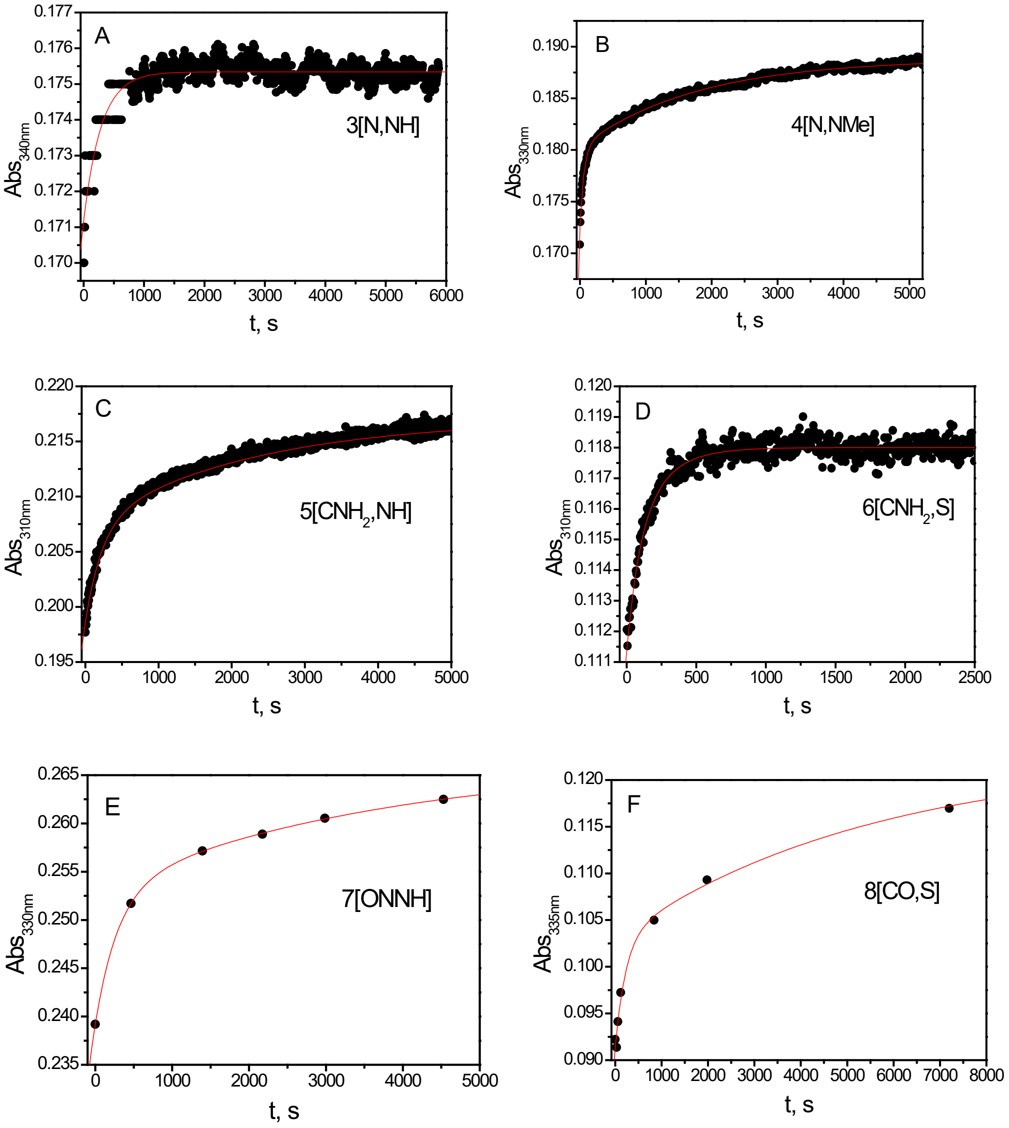

Figure S13. Biexponential kinetic curves of DNA with A) **3[N,NH]** $C_D = 1.03\times10^{-5}$ M, B) **4[N,NMe]** $C_D = 9.44\times10^{-6}$ M, C) **5[CNH₂,NH]** $C_D = 1.13\times10^{-5}$ M, D**) 6[CNH₂,S]** $C_D = 1.22\times10^{-5}$ M, E) **7[CO,NH]** $C_D = 2.36\times10^{-5}$ M, F) **8[CO,S]** $C_D = 9.40\times10^{-6}$ M. $C_P/C_D = 20$.

Table S10. Rate constants for the covalent binding between the complexes and DNA.

Complex	k_c x 10^3 s^{-1}
**1[C,NH]**	----
**2[C,NMe]**	----
**3[N,NH]**	20 ± 2
**4[N,NMe]**	16 ± 1
**5[CNH₂,NH]**	3.9 ± 0.1
**6[CNH₂,S]**	6.4 ± 0.2
**7[CO,NH]**	3.7 ± 0.1
**8[CO,S]**	6.0 ± 0.2

The formation of covalent DNA/Ir-complex product (PD), can be represented according to the following reaction:

$$P + D \overset{k_c}{\rightarrow} PD \qquad \text{(S1)}$$

where P represents the polynucleotide DNA and D the Iridium-metal complexes. Henceforth, C_D and C_P stand for the analytical D and P concentrations, respectively.

Nevertheless, we observed that for each complex, the k_c values remained essentially constant with the increment of C_P (Figure S9), proving that the reaction is monomolecular. Therefore, PD on equation 6 cannot be obtained in a unique step, since the reaction would be bimolecular and k_c would be C_p dependent. These results can be interpreted assuming the mechanism represented in equation 2: (i) the reaction between P and D leads to PD_I, being K_1 the equilibrium constant of this fast step, (ii) the PD_I complex turns into PD in a second irreversible and unimolecular step with rate constant, k_c, being the binding rate-determining constant.

$$\mathrm{P + D} \overset{K_1}{\rightleftarrows} \mathrm{PD_I} \overset{k_c}{\rightarrow} \mathrm{PD} \qquad \text{(S2)}$$

It is remarkably that k_c of **3[N,NH]** and **4[N,NMe]** the highest constants indicating the H_2O release from the coordination sphere is favored in N^N complexes, whereas in C^N we can ruled out a covalent binding to DNA.

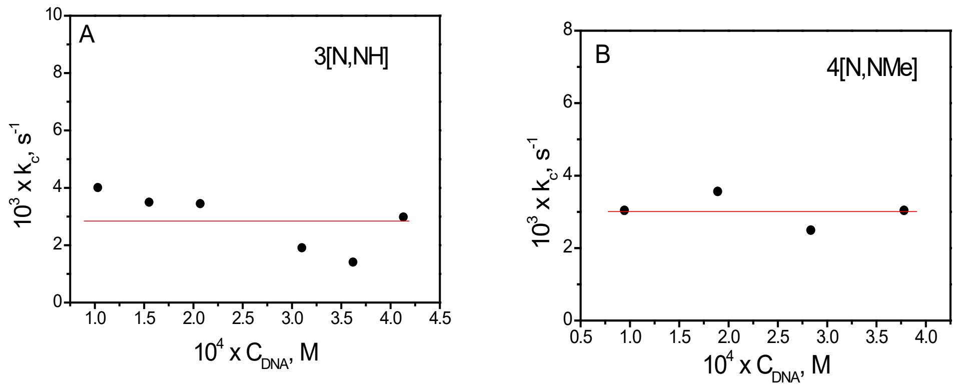

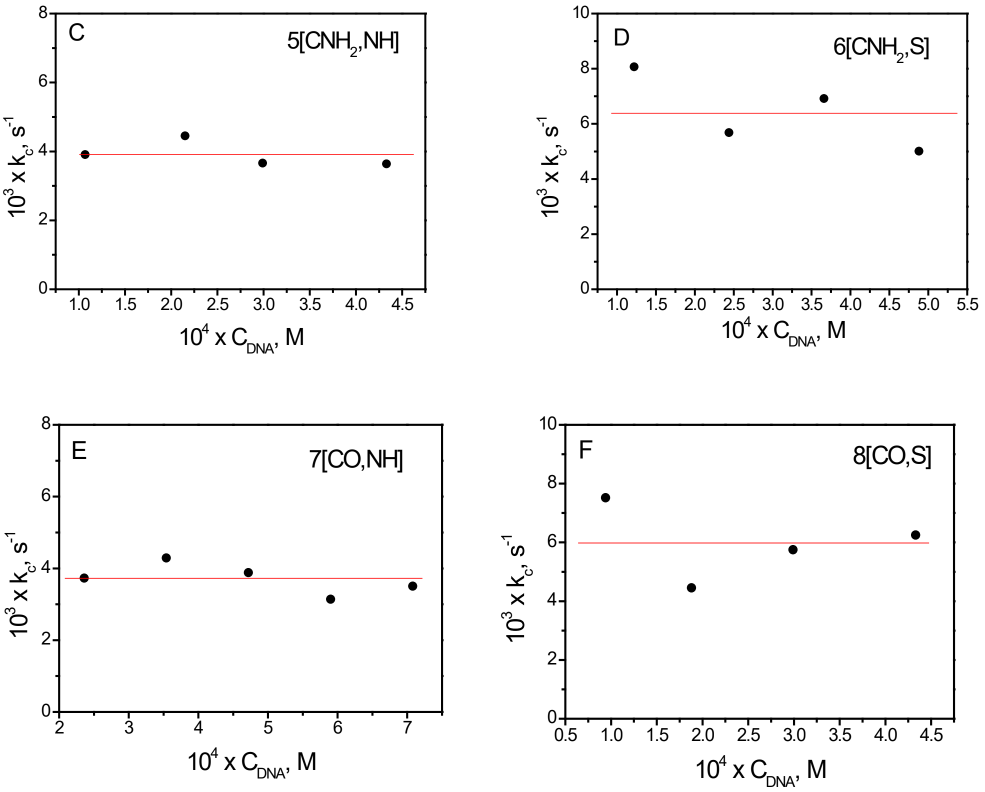

Figure S14. k_c versus C_P plot corresponding to formation of the DNA covalent binding with: A) **3[N,NH]** $C_D = 1.03\times10^{-5}$ M, B) **4[N,NMe]** $C_D = 9.44\times10^{-6}$ M, C) **5[CNH₂,NH]** $C_D = 1.13\times10^{-5}$ M, D) **6[CNH₂,S]** $C_D = 1.22\times10^{-5}$ M, E) 7**[CO,NH]** $C_D = 2.36\times10^{-5}$ M, F) **8[CO,S]** $C_D = 9.40\times10^{-6}$ M.

In order to confirm that the kinetic traces observed correspond to a covalent binding, NMR experiments with the 9MeG nucleobase and 5'-GMP nucleotide were performed. No interaction was observed between complex **1[C,NH]** and 9MeG in DMSO-d_6 letting us definitely discard DNA covalent binding. However, the spectrum of the reaction between 9MeG and complex **3[N,NH-Cl]Cl** in D_2O (see Figure 14) after 30 min exhibits resonances for **3[N,NH**] and a set of signals for a new product. The latter includes a singlet at 7.2 ppm attributed to proton H8 of coordinated 9MeG. This observation reveals that **3[N,NH]**, to give **3[N,NH-G]**$^+$, reaches a fast equilibrium.

Regarding the interaction with the nucleotide, the reaction between 5'-GMP and **3[N,NH]** was also studied by ^{1}H NMR in D_2O at 25 °C. Several spectra were recorded for a 5 mM solution of **3[N,NH]Cl** and 5'-GMP (5 mM, at pH 7). That is, a (1:1) stoichiometry. The reaction evolved really fast, and it was almost finished in 5 minutes. The spectra showed two different sets of signals, since two singlets appeared for the H8 proton of the 5'-GMP (see Figure S15A), as well as two singlets for the Cp*. The ^{31}P{^{1}H} NMR showed two intense peaks and two weaker signals among them (Figure S10B), being the most shielded the free 5'-GMP. In light of the previous results with 9MeG, and both the ^{1}H and the ^{31}P NMR experiments, we can conclude that the coordination of 5'-GMP to the metallic fragment of **3[N,NH]** occurs initially through the phosphate and then, through the N7 of the nucleobases.

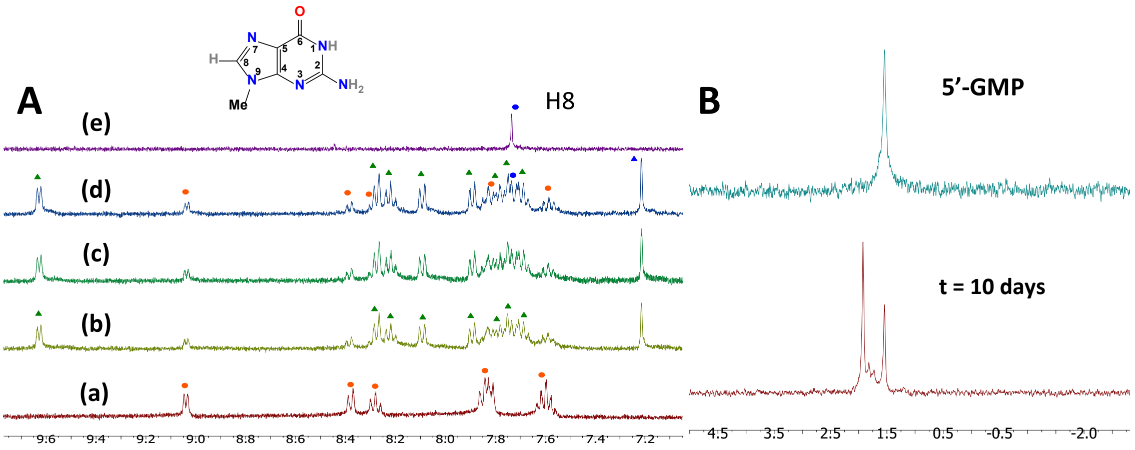

Figure S15. **A)** Evolution of the aromatic region in the ^{1}H NMR spectrum of **3[N,N-H$_2$O]**$^+$ (5 mM, pH 5, as a solid precipitated at pH 7) with 9MeG (5 mM, pH 7) in D$_2$O at 25 °C. The orange spots refer to the complex **3[N,NH]**, the blue ones to the free 9MeG, the green triangles refer to the new product **3[N,N-G]**$^+$ and the blue triangle to the coordinated 9MeG. (a) Spectrum of the complex before addition of 9MeG, (b) at t=5 min, (c) at t=10 min, (d) at t=30 min and (e) spectrum of free 9MeG at pH 7. **B)** ^{31}P{^{1}H} NMR spectra for the reaction of 5’-GMP (5mM, pH = 7) with 5mM of complex **3[N,NH]** recorded after 10 days. The upper spectra show the free 5’-GMP, and the lower the coordinated adducts.

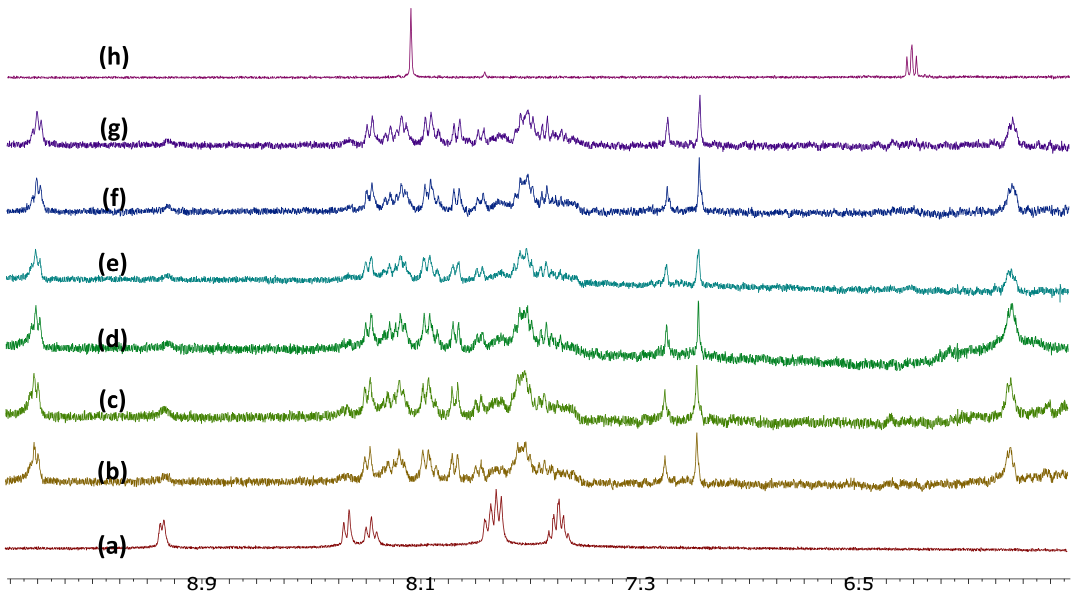

Figure S16. Evolution of the aromatic region of **3[N,NH]** with **5’-GMP** in D$_2$O at 25 °C. (a) Spectrum at t=0, (b) at t=5 min, (c) at t=25 min, (d) at t=1 h, (e) at t=4 h, (f) at t=22 h and (g) at t= 48 h. (h) Spectrum of **5’-GMP**.

The reaction of 5’-GMP with **5[CNH$_2$,NH]** was studied by NMR as well (Figure S15). In this case, the evolution of the spectra apparently displayed two sets of peaks (apart from those for the initial compound), although the aliphatic region showed a complex signal related to even four different products. However, one of them likely corresponds to the initial complex. The ^{31}P{^{1}H} NMR showed three peaks (see Figure S17), suggesting the formation of different products through different binding sites, and even their diastereoisomers.[3]

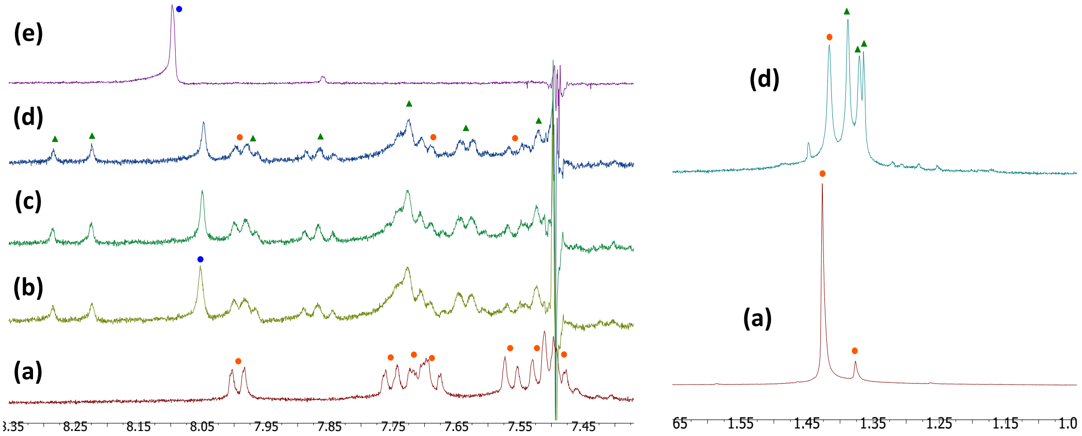

Figure S17. **A)** Evolution of the aromatic (left) and aliphatic (right) regions of the ^{1}H NMR spectrum **5[NH₂,NH]Cl** in the presence of 5'-GMP in D_2O at 25 °C. The orange spots illustrate the complex 5[NH₂,NH-H₂O]$^{2+}$, the blue ones the free 5'-GMP and the green triangles refer to the new product **5[CNH₂,NH-GMP]$^{2+}$**. (a) Spectrum at t=0, (b) at t=5 min, (c) at t=20 min, (d) at t=1 h. (e) Spectrum of 5'-GMP. **B)** $^{31}P\{^{1}H\}$ NMR spectra for the reaction of 5'-GMP (5mM, pH = 7) with 5mM of complex **5[NH₂,NH]Cl**.

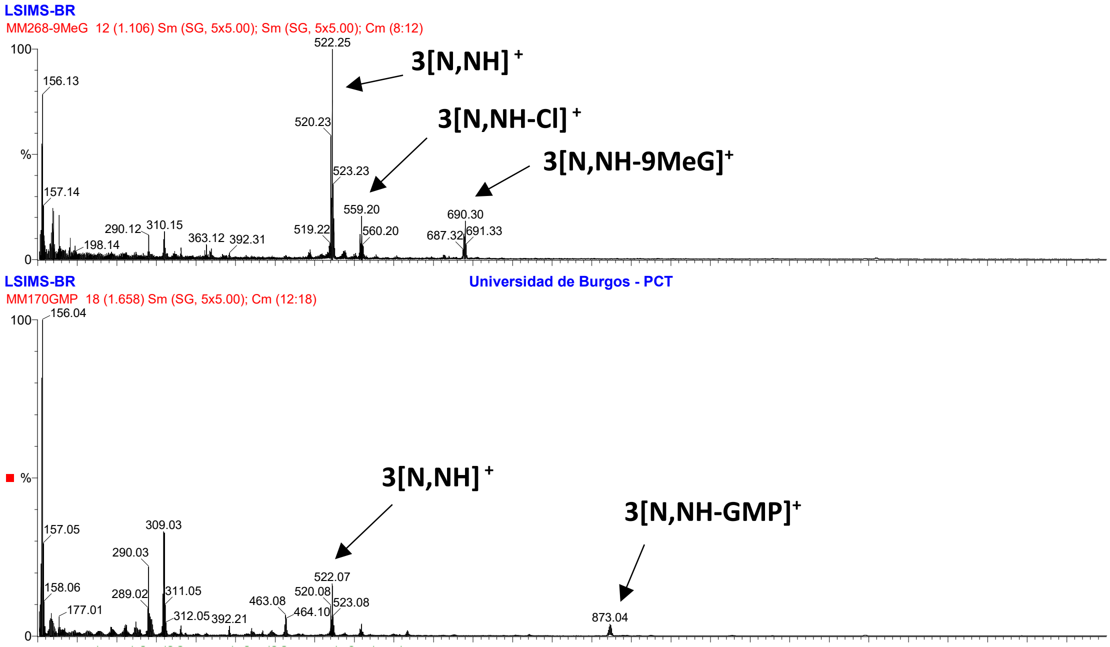

Figure S18. Mass spectra of **3[N,NH]** with 9-MeG (up) and 5'-GMP (down).

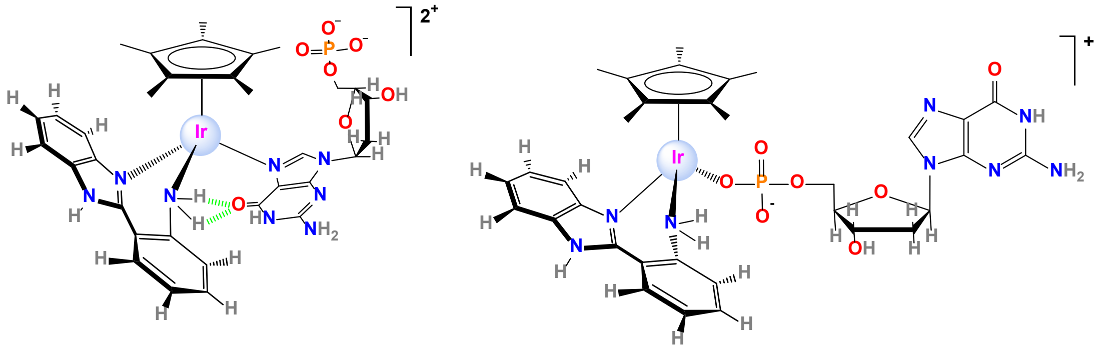

Figure S19. Products formed after reaction of **5[CNH₂,NH]** with 5'-GMP.

At this point it can be concluded that complexes **3[N,NH]**, **4[N,NMe]**, **5[CNH$_2$,NH]**, **6[CNH$_2$,S]**, **7[CO,NH]** and **8[CO,S]** are able to covalently bind DNA. By contrast, DNA covalent binding can be ruled out for **1[C,NH]** and **2[C,NMe].** To corroborate if these two complexes are able to bind DNA in a non-covalent way, absorbance titrations were carried out. No variation in the recorded spectra upon addition of DNA was observed (Figure S19).

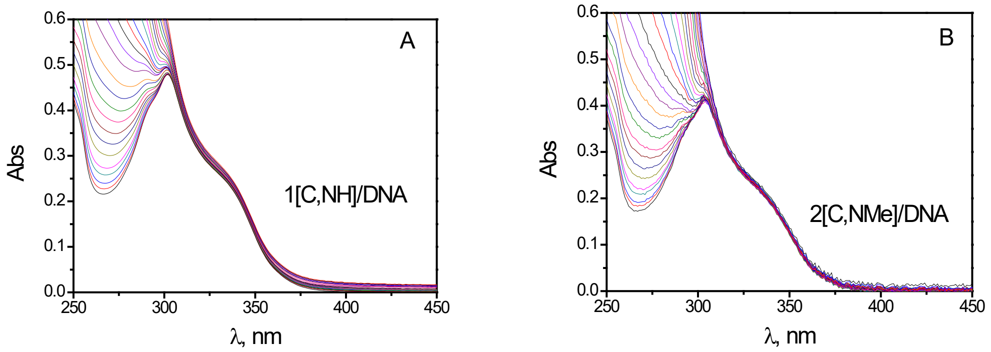

Figure S20. Absorbance titration for the A) **1[CNNH]**/DNA and B) **2[CNNMe]**/DNA systems.

Relative viscosity and melting temperature

The change of the contour length of DNA in the presence of the complexes provides relevant information about the binding. We did not observe any modification of DNA viscosity for **1[C,NH]** and **2[C,NMe]**, in agreement with the absence of interaction with DNA. In Figure S21 we can observe a prominent reduction of DNA length for **3[N,NH]**, **4[N,NMe]**, **5[CNH$_2$,NH]**, **6[CNH$_2$,S]**, **7[CO,NH]** and **8[CO,S]**, even at low C_D/C_P ratios, which is in agreement with bifunctional complexes. It is well-known that intercalation increases the viscosity,[4] the covalent binding slightly decreases it[5] and that strong decrease in viscosity can be attributed to groove binding, which, in contrast to intercalation, can shorten the helix by compaction.[6] That is, a bifunctional covalent-groove binding product is the most likely binding mode. In the case of **3[N,NH]**, the viscosity is drastically declined (Figure S21A), indeed, at $C_D/C_P = 0.3$ the viscosity is reduced by a half. Consequently, it would be expected a high cytotoxicity for **3[N,NH]**, due to its huge capacity to modify the DNA structure (see also Figure S21C). Nevertheless, as we will see below, its toxicity is low due its low cellular uptake.

An increase in the DNA melting temperature (T_m) indicates stabilization of the double helix structure. On the contrary, the decrease of T_m indicates destabilization. The variation of T_m in the presence of the Ir complexes at different C_D/C_P ratios is shown in Figure S22. No variations were observed for **1[C,NH]** and **2[C,NMe]**, confirming again no DNA interaction. According to melting studies complexes **3[N,NH]** , **4[N,NMe]** , **5[CNH$_2$,NH]** and **6[CNH$_2$,S]** show strong and very complex interaction with DNA. The differences in T_m point out the formation of more than one complex depending on the C_D/C_P ratio, as we observed in CD and viscosity too.

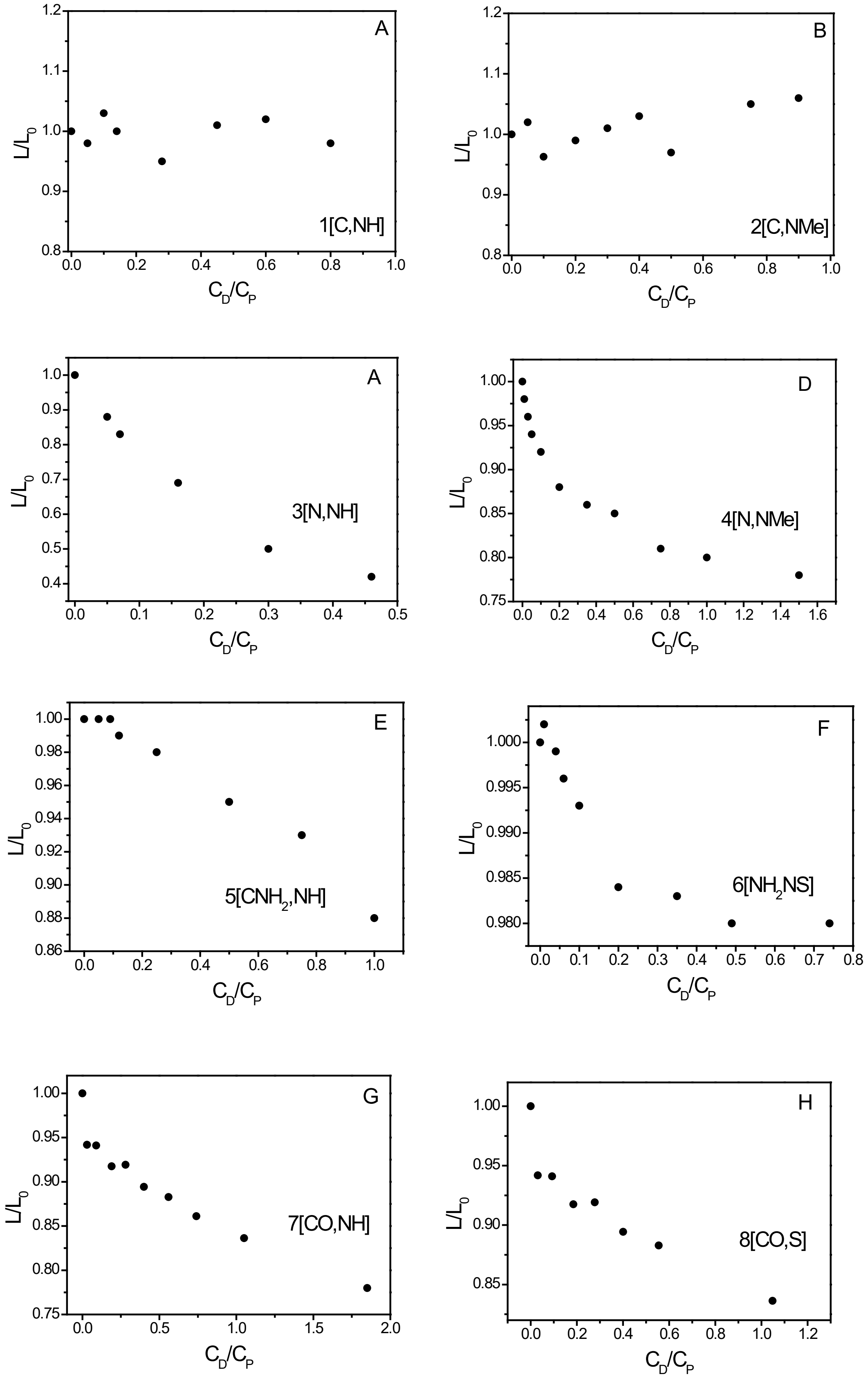

Figure S21. Relative elongation of DNA in the presence of A) **1[C,NH]**, B) **2[C,NMe]**, C) **3[N,NH]**, D) **4[N,NMe]**, E) **5[CNH$_2$,NH]**, F) **6[CNH$_2$,S]**, G) **7[CO,NH]** and H) **8[CO,S]** with DNA as a function of the C_D/C_P ratio. $C_P^0 = 3\times10^{-4}$ M.

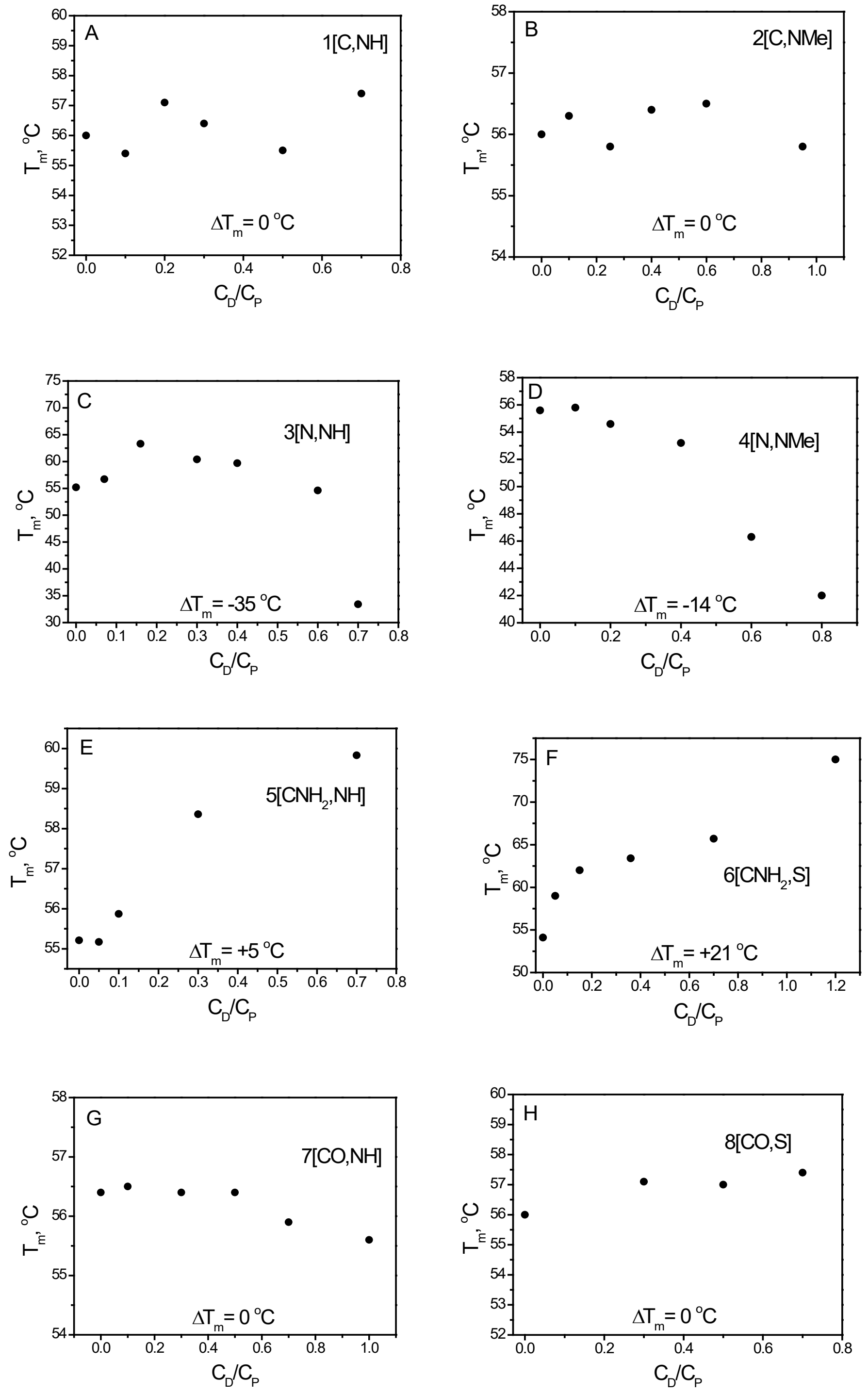

Figure S22. Melting temperature as a function of the C_D/C_P ratio of the systems A) **1[C,NH]**/DNA, B) **2[C,NMe]**/DNA, C) **3[N,NH]**/DNA, D) **4[N,NMe]**/DNA, E) **5[CNH_2,NH]**/DNA, F) **6[CNH_2,S]**/DNA, G) **7[CO,NH]**/DNA and H) **8[CO,S]**/DNA.

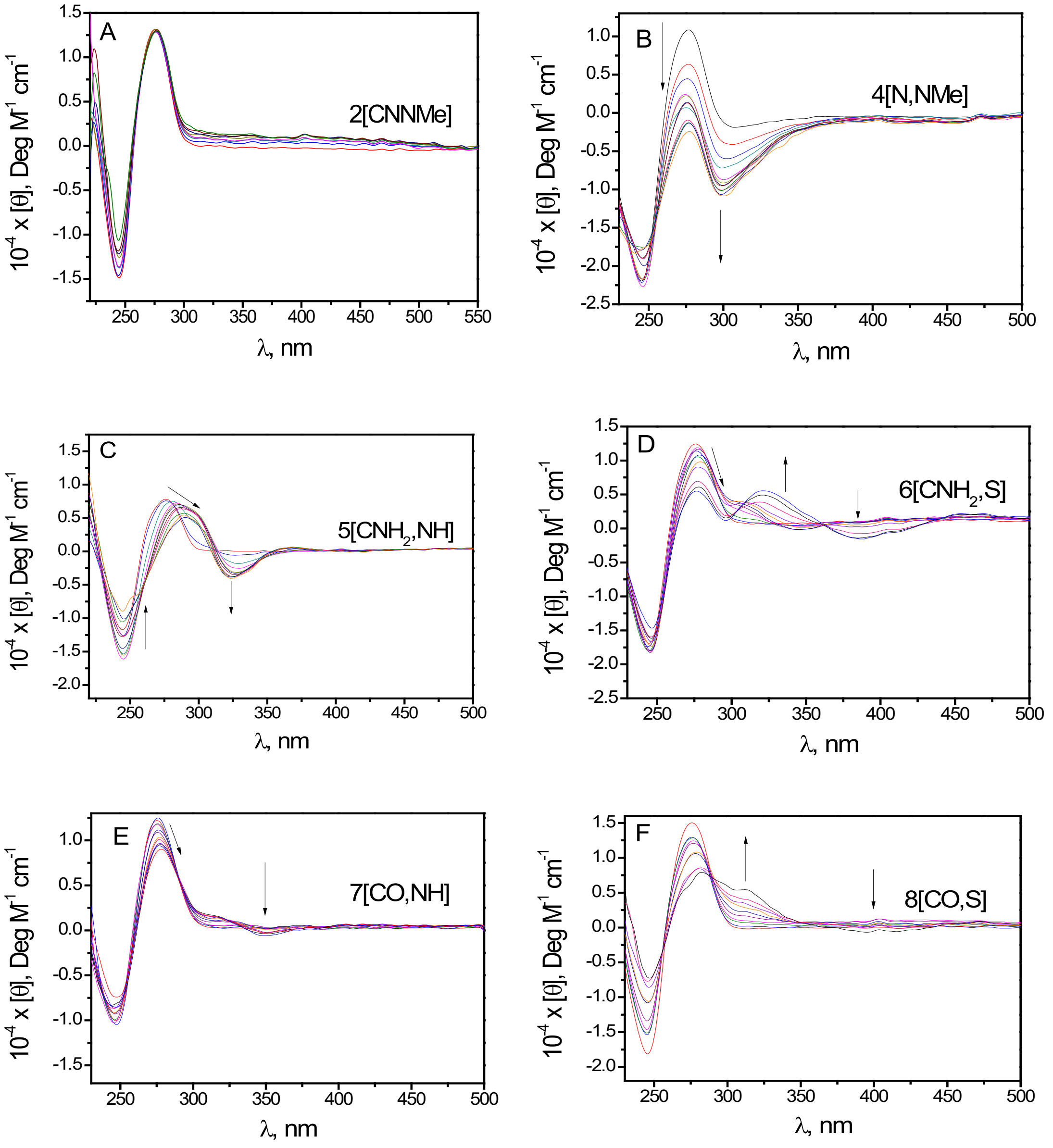

Figure S23. Circular dichroism spectra of DNA with A) **2[C,NMe]**, B) **4[N,NMe]**, C) **5[CNH₂,NH]**, D) **6[CNH₂,S]**, E) **7[CO,NH]** and F) **8[CO,S]**.

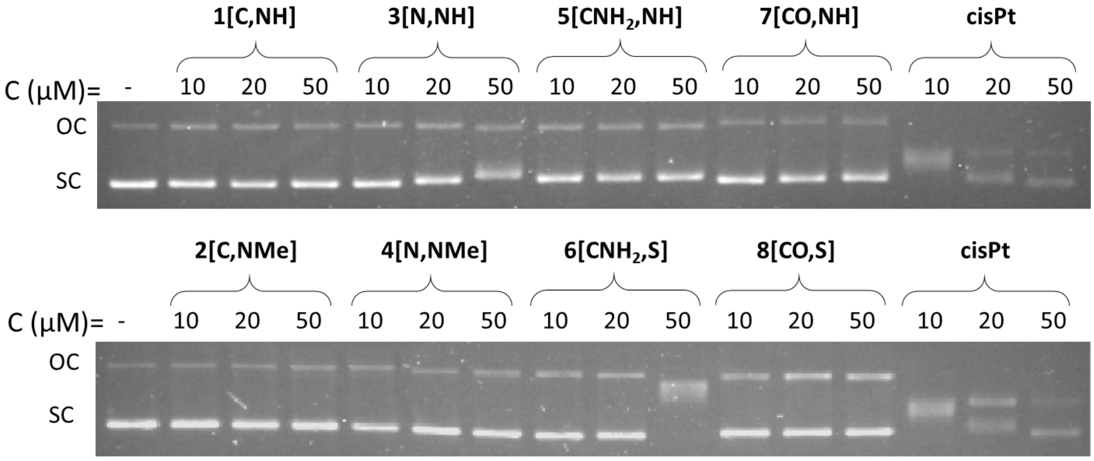

Figure S24**.** Electrophoretic mobility pattern of 20μM pUC18 plasmid DNA incubated overnight with different concentrations of the Ir complexes. cisPt is included for comparison.

3-Intracellular oxidative state

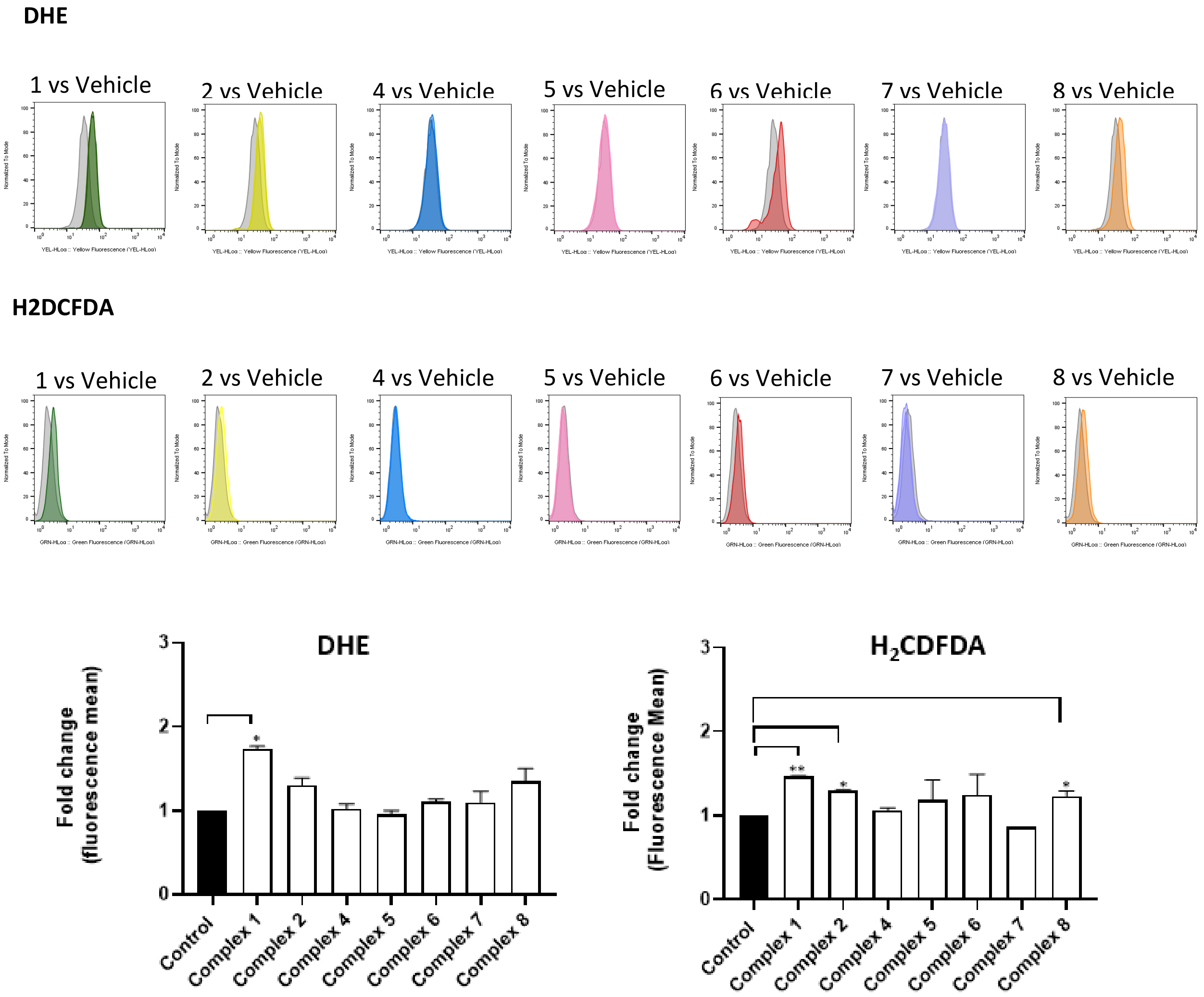

Figure S25. Oxidation of DHE and H_2DCFDA induced by indicated complexes and analysed by flow cytometry, 3 hours after treatment. Colored profile = Complex; Gray profile = Control. Images were representative of at least three independent experiments. The complex 3 was excluded of this study.

4-Biological effects of **1[C,NH]**.

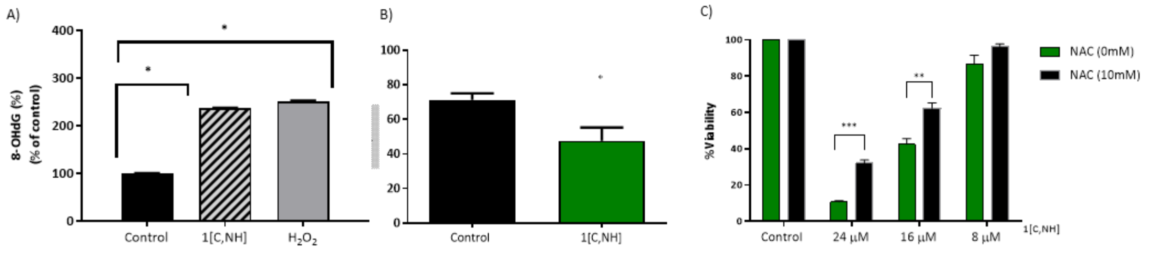

Figure S26. **A)** DNA damage levels measuring 8-hidroxi-2'-desoxiguanosina (8-OH-dG) using EpiQuik 8-OHdG DNA Damage Quantification Direct Kit. A549 cells were analyzed after 4 h incubation with 16 μM of **1[C,NH]**. Positive control (200 μM of H_2O_2). (* $p < 0.05$). B) GSH/GSSG levels measured using GSH/GSSG Glo Assay. A549 cells were analyzed after 1h incubation with **1[C,NH]**. Data shown are the mean and SD of at least two independent experiments. (** $p < 0.01$). **C)** Viability determined by MTT on A549 cells

treated at the indicated doses of **1[C,NH]** for 24 h. Previously cells were pre-incubated 2 h with NAC. Represented data are the mean and deviation of at least two independent experiments. (*** $p < 0.001$)

Movie S1.

Detection of Caspases 3/7

https://www.dropbox.com/s/4bs9ngl3uokct3d/Movie%20S.1.%20Cell%20Event%20Caspase%203-7%20detection.%20A549%20cells.mkv?dl=0

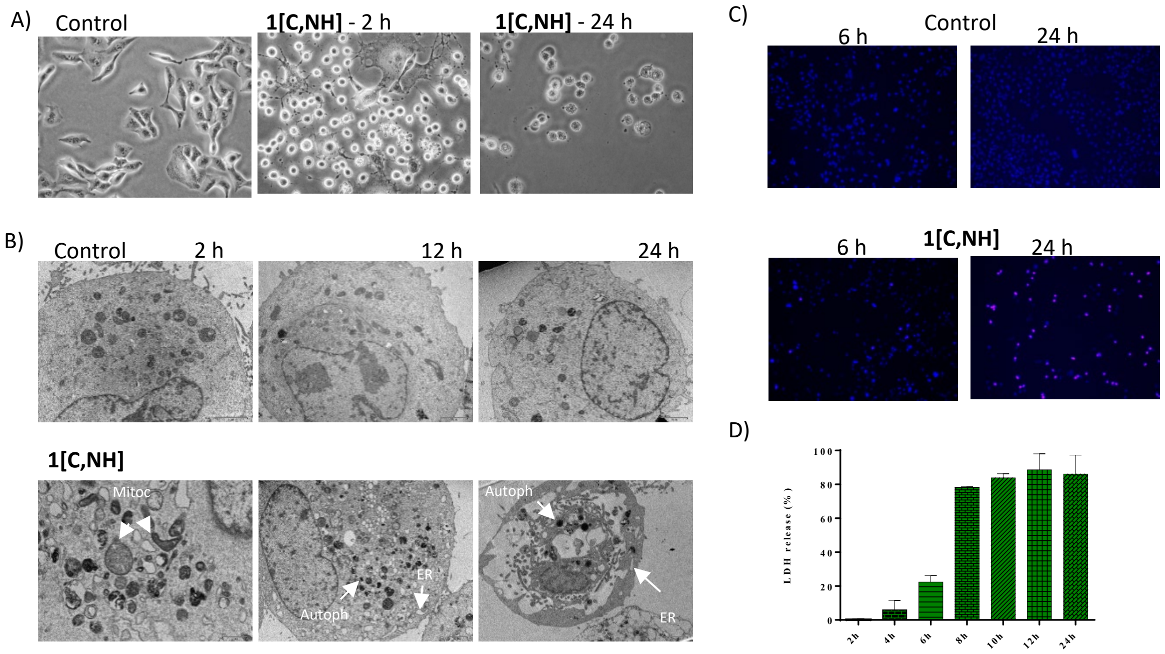

Figure S27. **A)** Images showing morphological changes of A549 cells after 2 h and 24 h contact with 16 µM of **1[C,NH]** compared to control. The fine white arrows show cells with apoptotic typical morphology, emission of prolongations, retraction of the cytoplasm, rounding, bubbling in the membrane, formation of apoptotic bodies, etc. After 2 h there are abundant apoptotic cells and after 24 h, in thick arrows cells with secondary necrosis. **B)** TEM images, A549 treated cells showed damage in mitochondria (Mitoc), dilated ER (ER) and autophagosomes (Autoph). Scale bar: 2µM. **C)** Images showing staining with the vital dyes Hoechst and IP, after 6 h and 24 h from the 16 µM administration of **1[C,NH]**. (10X Magnification). **D)** LDH release at indicated time of incubation with 16 µM of **1[C,NH]** in media supplemented with 1% FBS. (100 % represents LDH released after treatment with Triton X-100 that completely lysis the cells). Data shown are the mean and SD of at least three independent experiments.

Movie S2.

Cell viability

https://www.dropbox.com/s/i6nubxio4ybeje7/Movie%20S.2.%20Live%20%20Dead.%20A549%20cells.mkv?dl=0

Table S11. Differential gene Expression. Arrays data have been deposited in the ArrayExpress database at EMBL-EBI (www.ebi.ac.uk/arrayexpress) under accession number E-MTAB-8603.

Differential gene expression (1[C,NH]) vs. Control)		
**Genes**	**RT-qPCR ($2^{-\Delta\Delta Ct}$)**	**Microarray (Fold Change (linear))**
**Stress response**		
FOSB	> 100	>100
EGR1	> 100	>100
ATF3	> 100	37
DDIT3	7.8	23
PPP1R15A	14.8	11
CHAC1	3.5	3
**Apoptosis**		
BBC3	3.7	3.3
BCL2L11	3.6	5.1
PMAIP1	3.4	7.1
**Cell cycle**		
SKP2	0.3	-4.7
HIST1H1B	0.1	-6.6
**Other**		
MIR9-1	> 100	26.8
ATP1B2	19.5	34.8

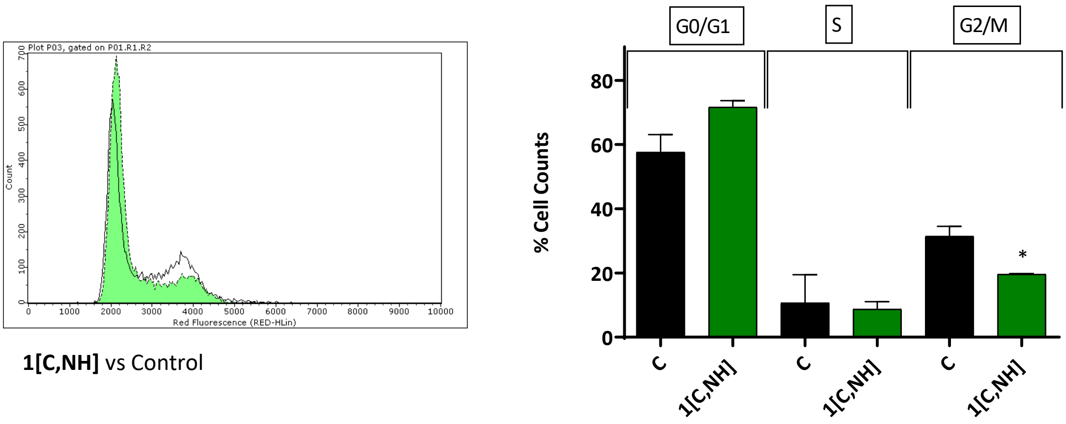

Figure S28**. Left:** Representative image of cell cycle distribution in A549 cells analyzed by flow cytometry. Cells were stained with PI after 24 h of treatment with 8 µM of **1[C,NH]** (green) and compared with control (white). **Right:** Cell cycle distribution graph. Data shown are the mean and SD of two independent experiments (C = Control; Ir **= 1[C,NH]**) (* $p < 0.05$).

Table S12.

Key parameters of mitochondrial respiration on A549 cells.

Group Name:	**Control**		**1[C,NH] - 16 µM**	
Parameters – OCR (pmol/min):	Mean	SD	Mean	SD
Non-Mitochondrial Oxygen Consumption	23.82	1.47	26.35	1.04
Basal Respiration	67.13	0.84	57.35	6.39
Maximal Respiration	101.69	11.71	47.24	14.31
Proton Leak	14.35	0.66	49.58	5.40
ATP Production	52.78	1.50	7.77	1.80
Spare Respiratory Capacity	34.57	12.55	0.27	0.54
Spare Respiratory Capacity as a %	151.76	19.16	80.93	19.49
Coupling Efficiency	78.65	1.31	13.65	2.64

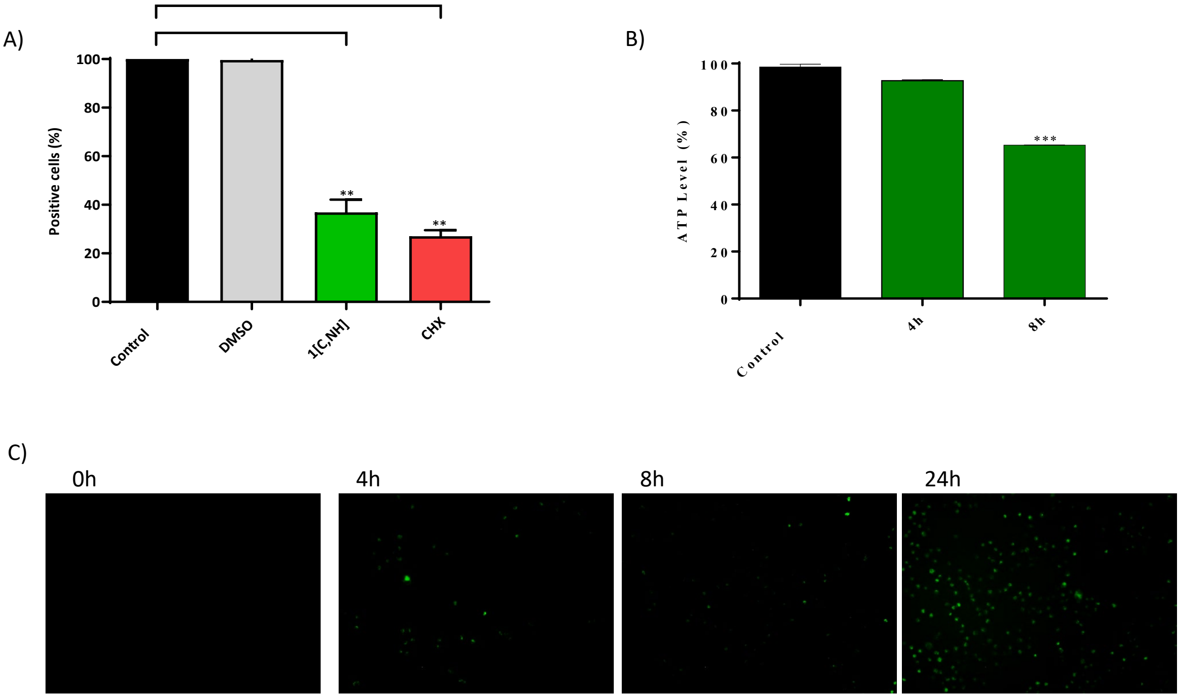

Figure S29. A) Protein synthesis was measured using Click-It Alexa Fluor-OPP on A549 cells after 6 h incubation with 16 µM of **1[C,NH]** or 500 µM of CHX (positive control). Represented data are the mean and deviation of at least two independent experiments. B) ATP levels after 4 h and 8 h incubation with 16 µM of **1[C,NH].** The graphs correspond to the average of at least two independent experiments. (*** $p < 0.001$). C) Determination of calcium accumulation in cytosol, by fluorescence microscope. Cells were pre-stained with Fluo-3AM. Images (10X) were captured at indicated incubation time with 16 µM of **1[C,NH]**.

Table S13

Key parameters of glycolysis on A549-gal cells.

Group Name:	**Control**		**1[C,NH] - 16 µM**	
Parameters – ECAR (mpH/min)	Mean	SD	Mean	SD
Non-Glycolytic Acidification	7.96	0.39	9.64	0.95
Glycolysis	16.56	0.23	44.81	1.56
Glycolytic Capacity	49.38	1.41	43.97	1.79
Glycolytic Reserve	32.82	1.22	2.48	2.00
Glycolytic Reserve %	307.80	0.07	98.26	0.04
Acute Response	1.94	0.12	4.17	0.28

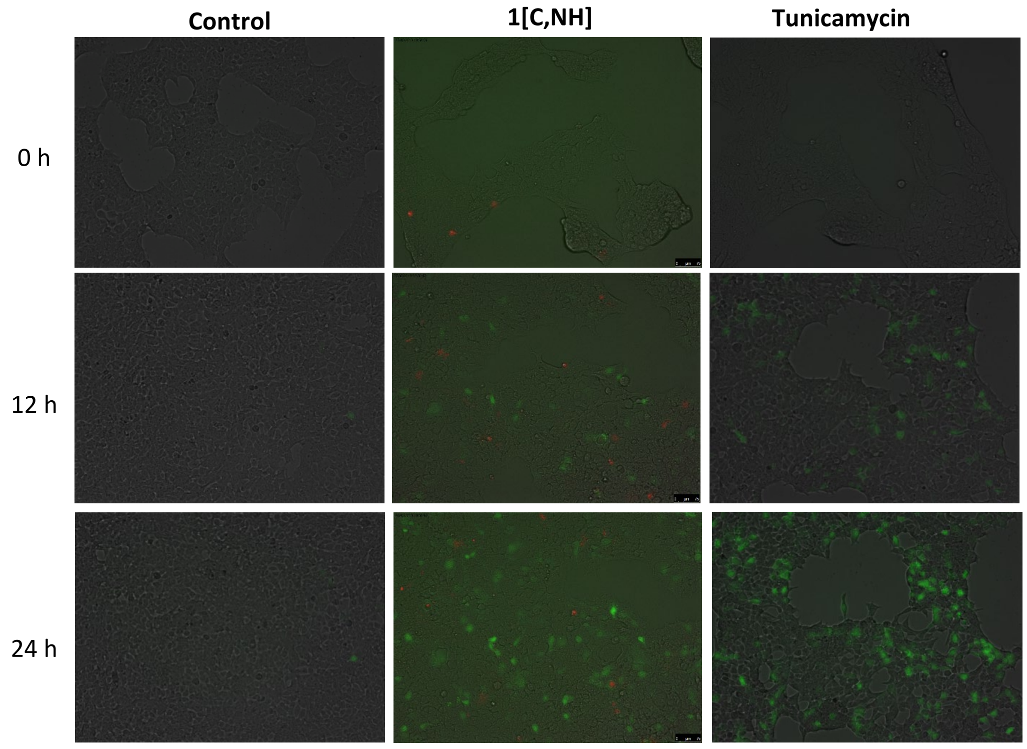

Figure S30**.** CHOP induction. HEK293 cells were transfected with the CHOP:: GFP plasmid, using the Dreamfect ™ Gold transfection reagent (OZ Bioscience). They were subsequently treated with **1[C,NH]** (9.7 µM). Tunicamycin (0.5 µM) was used as a positive control. Cells were stained with IP (1 µg / ml) to detect if there is death (red signal). The activity of said gene (green signal) was monitored by fluorescence microscopy. The emission of fluorescent green indicates the activation of the gene, which was detected in the cells treated with **1[C,NH]**.

5 – References

[1] F. Wang, H. Chen, S. Parsons, I. D. H. Oswald, J. E. Davidson, and P. J. Sadler, "Kinetics of aquation and anation of ruthenium(II) arene anticancer complexes, acidity and X-ray structures of aqua adducts.," Chem. - A Eur. J., vol. 9, no. 23, pp. 5810–5820, 2003.

[2] H. Chen, J. A. Parkinson, R. E. Morris, and P. J. Sadler, "Highly Selective Binding of Organometallic Ruthenium Ethylenediamine Complexes to Nucleic Acids: Novel Recognition Mechanisms.," J. Am. Chem. Soc., vol. 125, no. 1, pp. 173–186, 2003.

[3] N. Busto et al., "Anticancer activity and DNA binding of a bifunctional Ru(II) arene aqua-complex with the 2,4-diamino-6-(2-pyridyl)-1,3,5-triazine ligand.," Inorg. Chem., vol. 52, no. 17, pp. 9962–9974, Aug. 2013.

[4] G. Cohen and H. Eisenberg, "Viscosity and sedimentation study of sonicated DNA-proflavine complexes.," Biopolymers, vol. 8, no. 1, pp. 45–55, 1969.df

[5] M. N. Patel, C. R. Patel, H. N. Joshi, and K. P. Thakor, "DNA interaction and cytotoxic activities of square planar platinum(II) complexes with N,S-donor ligands.," Spectrochim. Acta, Part A Mol. Biomol. Spectrosc., vol. 127, pp. 261–267, 2014.

[6] B. Willis and D. P. Arya, "Triple recognition of B-DNA.," Bioorg. Med. Chem. Lett., vol. 19, no. 17, pp. 4974–4979, 2009.
