# Supplementary figures and images for: Targets, Mechanisms and Cytotoxicity of Half-Sandwich Ir(III) Complexes Are Modulated by Structural Modifications on the Benzazole Ancillary Ligand
Source: Cancers (Basel). 2022 Dec 24;15(1):107. doi: 10.3390/cancers15010107 (PMC9818021; doi:10.3390/cancers15010107)

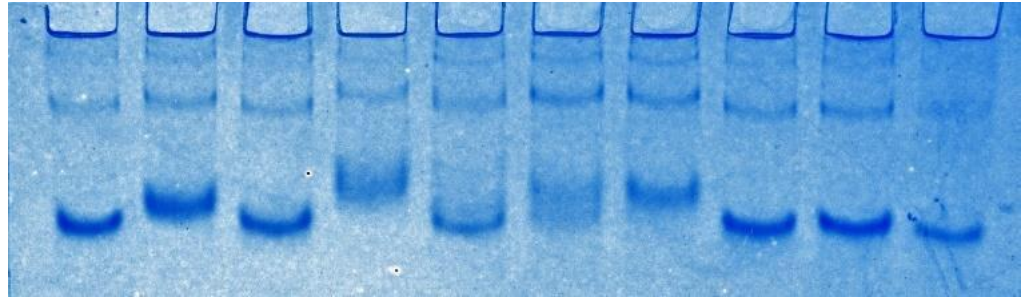

**Figure S31.** Original Western Blot for Figure 4C.

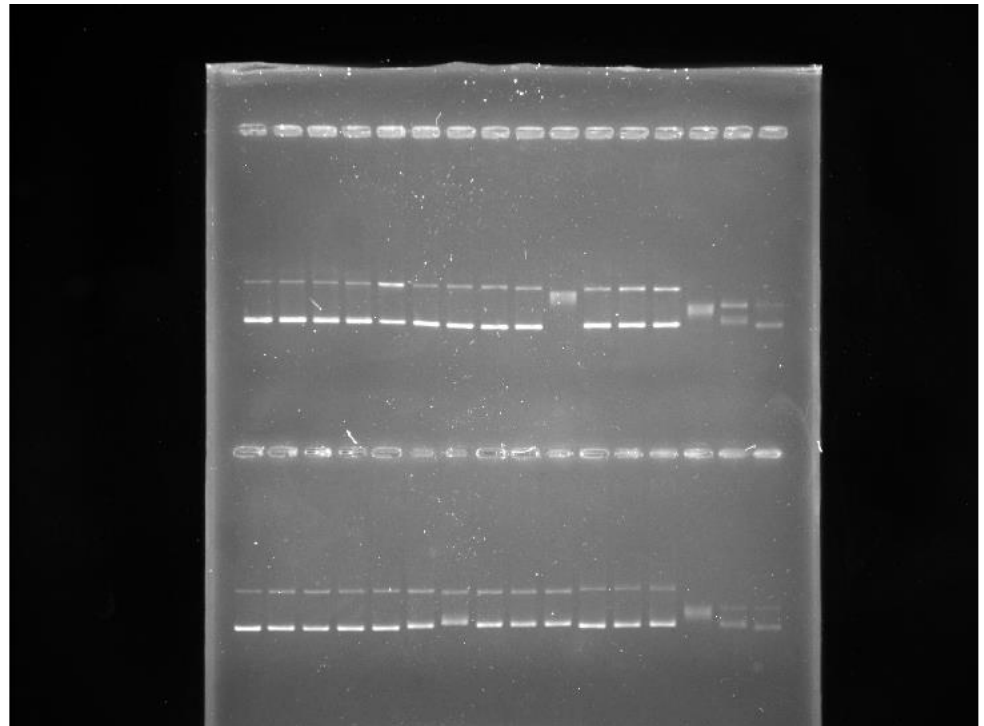

**Figure S32.** Original Western Blot for Figure S24.

Supplement: Supplementary file 1 [file cancers-15-00107-s001.zip › Figure S31 and S32.pdf]
